# Model-based driving mechanism analysis for butyric acid production in *Clostridium tyrobutyricum*

**DOI:** 10.1186/s13068-022-02169-z

**Published:** 2022-06-25

**Authors:** Jun Feng, Xiaolong Guo, Feifei Cai, Hongxin Fu, Jufang Wang

**Affiliations:** 1 School of Biology and Biological Engineering, South China University of Technology, Guangzhou, 510006 China; 2 Guangdong Provincial Key Laboratory of Fermentation and Enzyme Engineering, South China University of Technology, Guangzhou, 510006 China

**Keywords:** *Clostridium tyrobutyricum*, Butyrate, Genome-scale metabolic model, Metabolic driving forces, Energy conversion, Hydrogenase

## Abstract

**Background:**

Butyric acid, an essential C4 platform chemical, is widely used in food, pharmaceutical, and animal feed industries. *Clostridium tyrobutyricum* is the most promising microorganism for industrial bio-butyrate production. However, the metabolic driving mechanism for butyrate synthesis was still not profoundly studied.

**Results:**

This study reports a first-generation genome-scale model (GEM) for *C. tyrobutyricum*, which provides a comprehensive and systematic analysis for the butyrate synthesis driving mechanisms. Based on the analysis in silico, an energy conversion system, which couples the proton efflux with butyryl-CoA transformation by two redox loops of ferredoxin, could be the main driving force for butyrate synthesis. For verifying the driving mechanism, a hydrogenase (HydA) expression was perturbed by inducible regulation and knockout. The results showed that HydA deficiency significantly improved the intracellular NADH/NAD^+^ rate, decreased acetate accumulation (63.6% in serum bottle and 58.1% in bioreactor), and improved the yield of butyrate (26.3% in serum bottle and 34.5% in bioreactor). It was in line with the expectation based on the energy conversion coupling driving mechanism.

**Conclusions:**

This work show that the first-generation GEM and coupling metabolic analysis effectively promoted in-depth understanding of the metabolic driving mechanism in *C. tyrobutyricum* and provided a new insight for tuning metabolic flux direction in Clostridium chassis cells.

**Supplementary Information:**

The online version contains supplementary material available at 10.1186/s13068-022-02169-z.

## Introduction

Butyric acid is an essential C4 platform chemical, and its derivatives are widely used in food, chemical, pharmaceutical, cosmetic, and animal feed fields [1, 2]. The annual consumption of butyric acid is ∼80,000 tons [3]. For the field of animal feed, butyric acid derivatives, as feed additives, have been demonstrated to provide several benefits for improving gut health and protecting against harmful microbes [4]. With the limitation of antibiotics application in the animal husbandry industry, the butyric acid derivatives market will continually expand during the forecast period. At present, the chemical synthesis process, using petroleum-based products as feed-stocks, is the primary mode for butyric acid production [5]. With the increasing awareness of environmental issues and the need for sustainable development, many researchers focus on developing the bio-based butyric acid production process from renewable feed-stocks [6–9]. Among these studies,* Clostridium tyrobutyricum*, a Gram-positive, strictly anaerobic bacterium, is widely regarded as the most promising microbial cell factory for butyric acid production [10–13]. However, in *C. tyrobutyricum*, butyrate production was coupled with acetate production, carbon dioxide and hydrogen emission, and also cell growth. This co-producing characteristic, especially for the by-production of acetate, reduces the yield of butyric acid and increases the cost of the purification process. Thus *C. tyrobutyricum* has been engineered for improving the fermentation performance, such as blocking the acetate synthesis pathway (knockout of ack and pta genes) [14, 15], improving the rate-limiting step for glucose utilization (overexpression of *pfkA* and *pykA* genes) [16], strengthening the butyrate synthesis pathway (overexpression of *cat*1 and *crt* genes) [17]. Although these metabolic modifications have improved the butyrate yield (improving ~ 11–18%), they still did not change the characteristic of co-producing butyrate with acetate. Moreover, there was no profoundly study for coupling metabolic driving mechanisms in *C. tyrobutyricum*.

Several studies have reported that artificial electron carriers, such as methyl viologen and benzyl viologen, can simultaneously suppress H_2_ biosynthesis and improve acetate assimilation in the fermentation of *C. tyrobutyricum* ATCC 25755 [18–20]. However, it was still unclear how inhibition of H_2_ biosynthesis affects acetate metabolic pattern and what a metabolic coupling relationship is between the H_2_, acetate and butyrate production. For discerning these mechanisms, a comprehensive understanding of the metabolic networks and the cofactor turnover pattern is necessary. The genome-scale metabolic model (GEM) is a powerful tool for analyzing metabolic flux distribution and predicting potential metabolic engineering targets [21]. GEMs have been constructed and applied to metabolic network analysis in several Clostridium species [22–24]. Although the whole genome of *C. tyrobutyricum* ATCC 25755 has been sequenced [25], the GEM of *C. tyrobutyricum* ATCC 25755 has not been previously constructed.

In the present study, the first-generation GEM for *C. tyrobutyricum* ATCC 25755 (*i*CT583) was established for exploring the coupling metabolic driving mechanism. In silico analysis indicated that butyrate synthesis could be driven by coupling with an anaerobic energy conversion system. In this system, two redox loops of ferredoxin prompted metabolic coupling within butyrate synthesis, CO_2_ and H_2_ emission, and also proton translocation across the cell membrane (by proton pump and ATPase). The switch of proton efflux pattern from Hyd to RnfA-E could improve the acetate assimilation. To validate these hypotheses, a typical [FeFe]-hydrogenase gene (*hydA*) was perturbed by inducible regulation and knockout, respectively. The result showed inhibiting HydA expression could significantly decrease the acetate accumulation and improve the intracellular NADH/NAD^+^ ratio and the butyrate yield. These phenomena agree with the analysis in silico for energy coupling metabolic driving mechanism, and this mechanism will provide a new insight for metabolic engineering *C. tyrobutyricum*.

## Results and discussion

### Construction and refinement of the GEM for *C. tyrobutyricum*

**Fig. 1:**
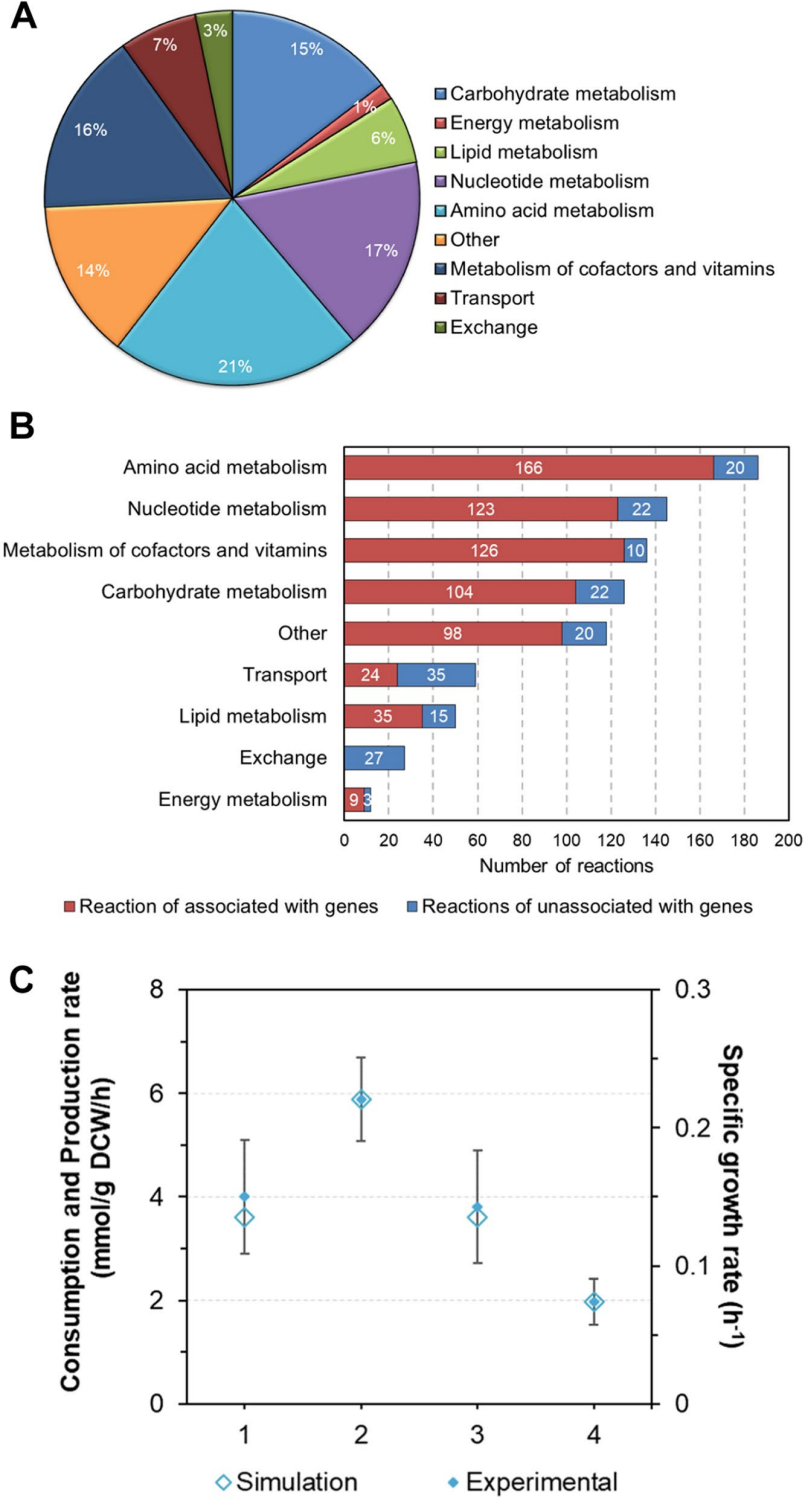
Refined characteristics of the GEM in *C. tyrobutyricum*. **A** Reaction distributions in each metabolic subsystem. **B** Number of reactions with gene or non-gene associations for different metabolic subsystems. **C** Comparison of the experimental and simulated metabolic flux distribution. The hollow diamond pattern represents the simulation where glucose was the carbon source with growth-associated maintenance (GAM) and non-GAM (NGAM) values of 35 and 5 ATP/g CDW/h, respectively. The diamond pattern represents the experimental data from fermentations in a 5-L bioreactor in this study

For the GEM construction for *C. tyrobutyricum* ATCC 25755, the genome sequence was re-annotated to associate with EC number, and then corresponding reactions were collected based on EC number (the model reactions list and construction process are presented in Additional file 1 and Additional file 2, respectively). Finally, the GEM of *i*CT583 contained 858 reactions, 798 metabolites, and spanned 583 genes. All reactions were assigned to nine metabolic subsystems (Fig. 1A), and they were further divided into 75 pathways based on the KEGG pathway system (Additional file 1, Pathway). In each subsystem, gene-associated reactions were dominant, except for transport and exchange reactions (Fig. 1B). By comparing with the GEMs for *C. acetobutylicum*, *C. beijerinckii* and *C. kluyveri* (Table 1), the total number of reactions in *i*CT583 (858 reactions) is not bigger, which could attribute to fewer open reading frames (ORFs) in *C. tyrobutyricum*. However, the ORF coverage rate of *i*CT583 (18.2%) is better than most of the clostridia GEMs in Table 1, except for the* i*Cac967.

**Table 1:** Comparison of different GEMs for different Clostridia

Strain	*C. cellulolyticum*	*C. beijerinckii*	*C. acetobutylicum*	*C. thermocellum*	*C. kluyveri*	*C. ljungdahlii*	*C. tyrobutyricum*
Total genome size^a^ (Mb)	4.1	6.0	4.1	3.8	4.5	4.6	3.1
Open reading frames (ORFs)	3488	5100	3748	3236	4422	4358	3180
Genome-scale model	*i*FS431	*i*CM925	*i*Cac967	*i*Cth446	*i*CKL708	*i*HN637	*i*CT583
Genes	431	925	967	446	708	637	583
Reactions	621	938	1231	660	994	785	858
Metabolites	603	881	1058	599	804	698	798
ORF coverage (%)	12.4	18.1	25.8	13.8	16.0	16.0	18.3
Reference	[45]	[23]	[46]	[47]	[48]	[49]	This study

^a^Data from the NCBI database

After preliminary construction, two evaluation modes were used to test the model at the qualitative and quantitative levels. For qualitative testing, 11 carbon and 2 nitrogen sources tested in vivo were selected to validate the integrity of the metabolic pathway in the GEM. As shown in Table 2, in the draft model, arabinose and glycerol as carbon sources and urea as nitrogen sources could not be utilized in silico owing to no gene-associated reactions for arabinose catabolism and transporter reaction for glycerol and urea. Therefore, based on the feature of *C. tyrobutyricum* in vivo, some reactions were supplemented, and the refined model could reach corresponding metabolic characteristics in silico (Table 2). For quantitative evaluation, the data from batch fermentation in a 5-L bioreactor were used as constraining conditions. The values for NGAM and GAM were preliminarily set as 5 and 40 mmol ATP/g CDW/h according to the model of *C. acetobutylicum* [24]. In this condition, the specific cell growth rate in silico (Additional file 1: Table. S1) was calculated as 0.17 h^−1^. Then, by further regulating GAM value to 35 mmol ATP/g CDW/h, the specific growth rate could be improved to 0.18 h^−1^, which was very close to the logarithmic phase-specific growth rate in vivo 0.20 ± 0.05 h^−1^ (Fig. 1C and Additional file 1: Table. S1). To examine whether the dataset we adopted was specific to the model, this study compared the specific cell growth rate with the data from other literature. As shown in Additional file 1: Table. S1, the specific growth rates in vivo ranged from 0.15 ± 0.02 to 0.28 ± 0.03 h^−1^, and our experimental data and fitted values matched well within this range. It demonstrated that the coefficients of the biomass compositions and the values of NGAM and GAM in the GEM were appropriate for fitting to cell growth and end-product distribution.

**Table 2:** Comparison of carbon and nitrogen utilization between in vivo and in silico data^a^

Substrates	In vivo	In silico growth in draft model	In silico growth in final model	Reference
Carbon sources				
Fructose	+	+	+	[50]
Glucose	+	+	+	[51]
Mannitol	+	+	+	[52]
Mannose	+	+	+	[53]
Galactose	−	-	-	[54]
Xylose	+	+	+	[55]
Arabinose	+	-	+	[56]
Glycerol	+	-	+	[57]
Sucrose	−	-	−	[58]
Maltose	−	−	−	[59]
Lactose	−	−	−	In this study
Nitrogen sources				
Ammonium	+	+	+	[60]
Urea	+	−	+	[61]

^a^Total 11 carbon sources and 2 nitrogen sources were selected from an experiment for validating the model. Glucose was used as the sole carbon source in simulating growth with nitrogen sources. + indicates available, −indicates unavailable

### In silico analysis of the coupling metabolic relationship

**Fig. 2:**
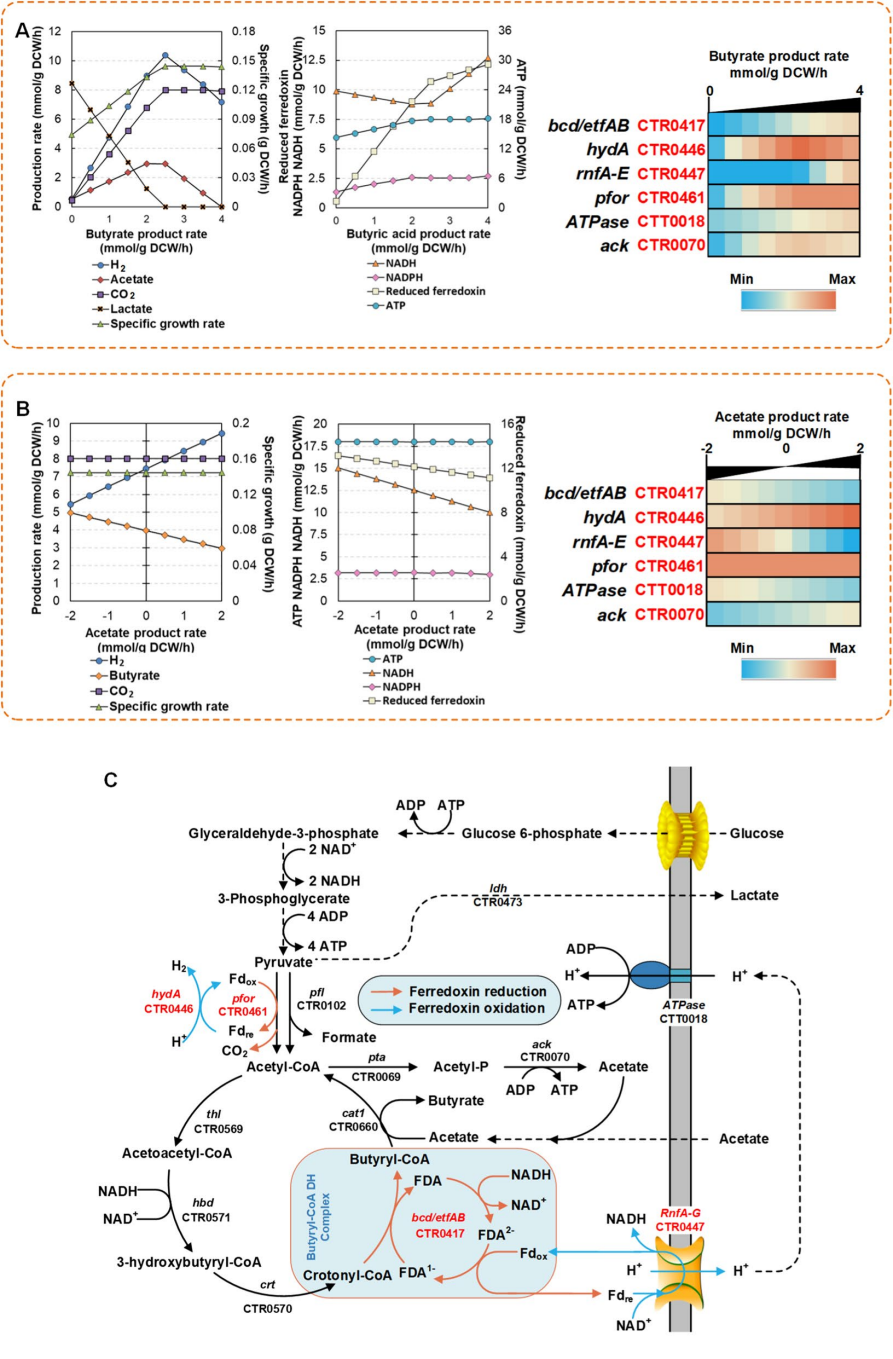
In silico analysis of the coupling metabolic relationship and acetate assimilation metabolic pattern in *C. tyrobutyricum*. **A** The effects of increased the specific butyrate production rate on the metabolic characteristics. The changes in specific production rates for the major product (left), the turnover rates of NADH, ATP, NADPH, and reduced ferredoxin (middle), and the key reaction fluxes (right) are shown. **B** The effect of regulating the specific acetate production rate on the metabolic characteristics (a negative value for the specific acetate production rate indicates acetate assimilation and a positive value indicates production). The changes in production rates for the major product (left), turnover rates of NADH, ATP, NADPH, and reduced ferredoxin (middle), and the key reaction fluxes (right) are shown. **C** Schematic of the coupling metabolic pathways for butyrate synthesis. Some metabolic pathways are labeled with the corresponding gene names and reaction IDs. Gene name and abbreviation: *ack*: acetate kinase; *bcd*: butyryl-CoA dehydrogenase; *cat*1: butyryl-CoA/acetate-CoA transferase; *crt*: crotonase; *etf*: electron-transferring flavoprotein; *hbd*: β-hydroxybutyryl-CoA dehydrogenase; *hydA*: hydrogenase; *ldh*: lactate dehydrogenase,* pta*: phosphotransacetylase;* pfor*: pyruvate: ferredoxin oxidoreductase; *pfl*: pyruvate formate-lyase; *thl*: thiolase; ATPase: F_o_F_1_ ATPase; RnfA-E: Rnf complex

For exploring the coupling metabolic relationship in silico, the specific butyrate production rate was set from 0 to 4 mmol/g CDW/h. Then the specific production rates of H_2_, CO_2_, acetate, lactate, and the specific growth rate were extracted to analyze the coupling relationship with butyrate production rate. As shown in Fig. 2A left, H_2_, CO_2_, acetate production and cell growth showed a positive coupling relationship with the specific butyrate production from 0 to 2 mmol/g CDW/h. In contrast, lactate production showed a negative coupling relationship in this range. When the specific butyrate production rate further increased (from 2.5 to 4 mmol/g CDW/h), the H_2_ and acetate production were negatively correlated with butyrate production. Moreover, butyrate production has little impact on the cell growth and CO_2_ emission. These results indicated that *i*CT583 could model the coupling metabolic feature in vivo. This coupling characteristic could be used as foundation for in silico analysis of the metabolic flux distribution and the cofactor turnover mode; whereas, at the high butyrate production level, the negative coupling relationship could attribute to the competitive carbon flux and reducing power consumption for butyrate synthesis.

Based the coupling characteristic in silico, the analysis of the cofactor turnover rates (Fig. 2A, middle) indicated that the turnover rate of reduced ferredoxin exhibited a marked positive coupling relationship with the increased butyrate production. Subsequently, some flux changes involving redox ferredoxin reaction in the model were extracted and analyzed. In these reactions, it formed two redox loops of ferredoxin, composed of pyruvate synthase (CTR0461) and hydrogenase (CTR0446), butyryl-CoA dehydrogenase/the electron transport flavoprotein complex (CTR0417) and RnfA-E (CTR0447) (Fig. 2C). Within the two redox loops, the flux of these reactions showed an increased trend with the increased specific butyrate production rate (from 0 to 2 mmol/g CDW/h). Furthermore, these reactions were also involved in butyrate production, H_2_ and CO_2_ the emission, and proton efflux from intracellular to extracellular (Fig. 2C). Therefore, the two redox loops could be vital for establishing the coupling metabolic characteristics. In addition, when the specific butyrate production rate increased to a high level (from 2.5 to 4 mmol/g CDW/h), a switch of the proton efflux pattern from Hyd to RnfA-E was also observed, and it also corresponded to an increase in the NADH turnover rate (Fig. 2A, middle and right). Correspondingly, the turnover rate and the reaction catalyzed by ATPase (CTT0018) showed an increasing trend with an increase in the specific butyrate production rate (Fig. 2A, middle and right). Together, these results demonstrated that the two redox loops of ferredoxin could convert the free energy of the redox reaction into proton motive force (PMF) by coupling proton efflux (H_2_ emission and proton pump). Then the PMF drove ATP synthase to produce ATP for cell growth, which created an anaerobic energy conversion system (Fig. 2C), whereas butyrate production and CO_2_ and H_2_ emission were more likely to be metabolic characteristics derived from this energy conversion system. Because this energy conversion system intrinsically converted electrochemical potential (such as PMF) to redox potential carried by organic redox couples (such as NADH/NAD^+^ and reduced/ oxidized ferredoxin), then the turnover of these organic redox couples further converted redox potential to some intermediate metabolites for cell growth and energy currency (some biochemicals can couple exergonic and endergonic reactions in cell metabolism, such as ATP and reduced ferredoxin) [26, 27]. In the turnover process of these organic redox couples, some enzymatic reactions coupled with each other to form the redox equivalent balance. Simultaneously, some metabolites would be generated as a kind of derived metabolic characteristics (in our cases, such as butyrate production and CO_2_ and H_2_ emission). It also explained the coupling relationship between cell growth and butyrate production since energy currency and some intermediate metabolites were necessary for cell growth. Therefore, it was believed that the energy conversion could be an original driving force for butyrate production in *C. tyrobutyricum*.

In the two redox loops of ferredoxin, the reaction mechanism of Bcd/EtfAB (CTR0417) and Hyd (CTR0446) was the critical factor for the coupling metabolic relationship. During *i*CT583 construction, The Bcd/EtfAB was considered as flavin-based electron bifurcation (FBEB). In the *C. tyrobutyricum* ATCC 25755 genome, two bcd genes (CTK_C26200 and CTK_C26360) were found, and the flanking region of CTK_C26360 contained two genes encoding electron transport subunits (*etfA* and* etfB*) (Additional file 2: Fig. S1). The gene arrangement is similar to *C. acetobutylicum* [28] and *C. kluyveri* [29]. In *C. kluyveri*, the Bcd/EtfAB complex has been reported to be an electron bifurcation reaction mechanism in which NADH as the electron donor drives the conversion of crotonyl-CoA to butyryl-CoA and the reduction of oxidized ferredoxin [29]. Therefore, the Bcd/EtfAB complex in *C. tyrobutyricum* ATCC 25755 is more likely involved in a similar reaction mechanism, whereas the reaction catalyzed by Hyd was considered as only ferredoxin-dependent model. There are three Hyd coding genes (CTK_C05160, CTK_C26290, and CTK_C 26580) in the *C. tyrobutyricum* ATCC 25755 genome. None of their flanking regions contains HydB and HydC coding gene, which are the necessary subunits for Hyd to perform electron bifurcation [30]. It demonstrated that these Hyds in *C. tyrobutyricum* ATCC 25755 belong to G1-group Hyds, which only use ferredoxin as the electron donor for H_2_ production [30]. Therefore, the configuration of these genes and the reaction mechanism in the *C. tyrobutyricum* genome provides solid evidence for the two redox loops of ferredoxin.

### In silico analysis of metabolic targets affecting the acetate metabolic pattern

In coupling metabolic analysis, acetate production showed a negative effect on a high level of specific butyrate production rate (from 2 to 4 mmol/g CDW/h). To further analyze the critical factor affecting acetate metabolism, the specific acetate production rate was set from − 2 to 2 mmol/g DCW/h (negative value indicates uptake) in *i*CT583. Then the variations in the end-products and cofactor turnover rates were extracted to analyze the corresponding relationships. With an increase in the specific acetate production rate, the specific butyrate production rate and H_2_ emission rate showed negative and positive coupling relationships, respectively (Fig. 2B, left). However, the cell growth and CO_2_ emission showed no obvious change. For the cofactors, only the NADH and reduced ferredoxin turnover rates showed a negative coupling relationship with the specific acetate production rate (Fig. 2B, middle). Checking the redox loop of ferredoxin, two sets of reactions (CTR0446/CTR0447 and CTT0018/CTR0070) showed a flux switching during the specific acetate production rate changing from -2 to 2 mmol/g DCW/h (Fig. 2B, right and Fig. 2C). This flux switching was similar to the situation at the high level of specific butyrate production rate (2.5 to 4 mmol/g CDW/h) (Fig. 2A, left). The switching of CTT0018/CTR0070 demonstrated that the energy production pattern was switched from Ack to ATPase, and CTR0446/CTR0447 switching meant that the proton efflux pattern was switched from Hyd to RnfA-E (Fig. 2C). Since the proton efflux was the premise for establishing PMF, PMF then can push ATPase to complete energy conversion. The proton efflux pattern switching can lead to the energy production pattern switching. Therefore, the proton efflux pattern switching from Hyd to RnfA-E could be a key incentive for inducing acetate assimilation. To verify this hypothesis, the flux for Hyd (CTR0446) and RnfA-E (CTR0477) was set from 0 to10 mmol/g DCW/h, and the specific acetate production rate was monitored. As expected, the increased Hyd flux had a negative effect on the acetate assimilation rate, while increased RnfA-E flux could improve the acetate assimilation (Additional file 2: Fig. S2). These results indicated that the proton efflux pattern switching could change the acetate metabolic pattern. As previously reported, adding methyl viologen or benzyl viologen into the fermentation system of *C. tyrobutyricum* ATCC 25755 could significantly inhibit H_2_ emission and induce acetate assimilation. Based on in silico analysis, methyl viologen or benzyl viologen could act as an inhibitor of Hyd or an activator of RnfA-E, which can change the electron transfer mode and then affect the metabolic characteristic. Therefore, either activation of RnfA-E or inhibition of Hyd could be a promising strategy for inducing acetate assimilation or reducing the acetate production.

### Induced regulation of hydA expression based on a theophylline-dependent riboswitch

**Fig. 3:**
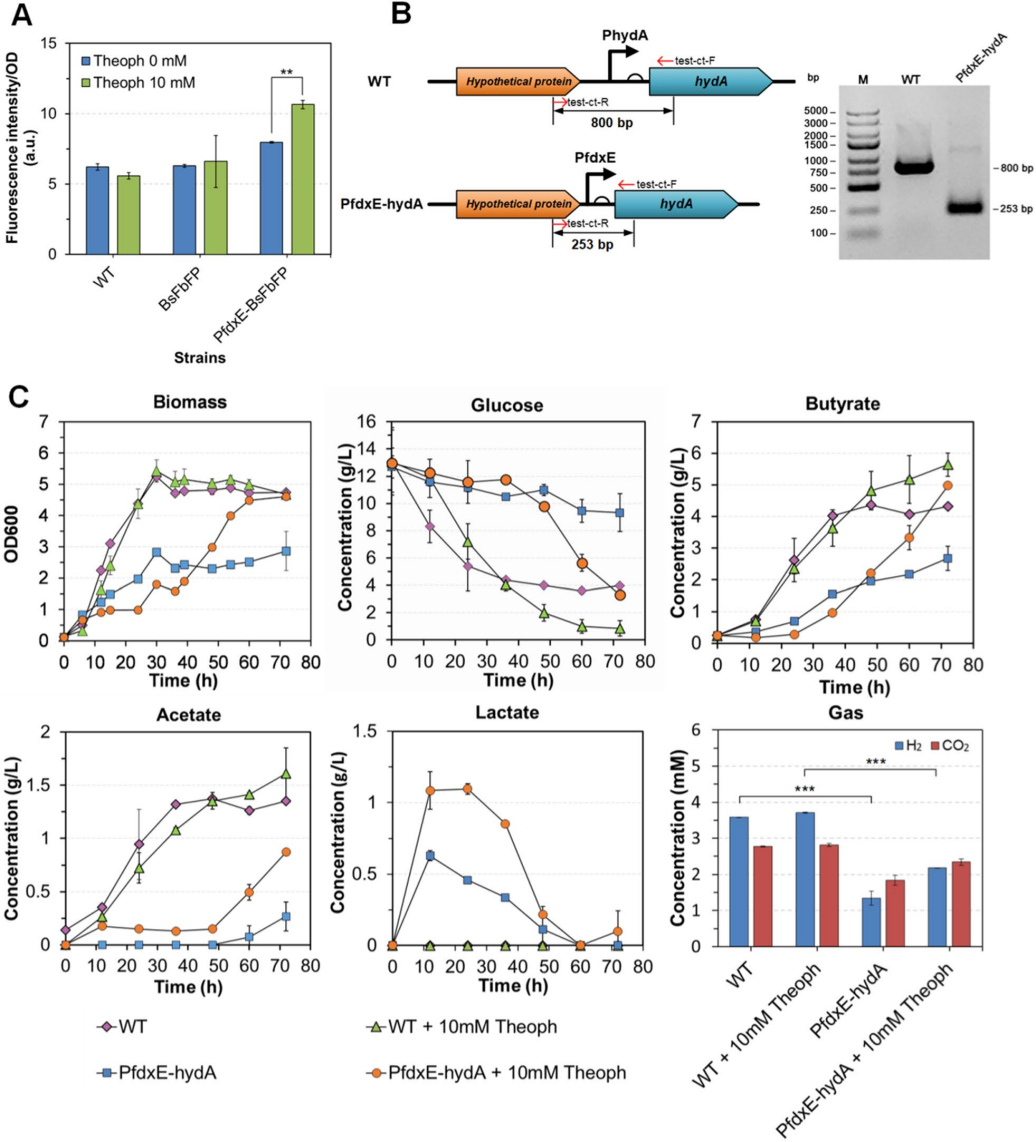
Effect of the inducible *hydA* expression on the metabolic characteristics. **A** Performance of a theophylline-dependent inducible expression motif in *C. tyrobutyricum*. The values represent averages for three replicates and the error bars represent the standard errors of these experiments (*p ≤ 0.05; **p ≤ 0.01; ***p ≤ 0.001, t test). **B** Verification of the replacement of the *hydA* promotor with a theophylline-dependently inducible expression motif. **C** Metabolic characteristics of the PfdxE-hydA strain under CGM without CaCO_3_ as a pH buffer; the values represent data from two replicates and the error bars represent the standard errors of these experiments

To verify the energy conversion system and the effects of regulating the electron transfer mode, this study plans to regulate Hyd expression and investigate the effects on the products distribution. As mentioned above, three Hyd coding genes were found in the *C. tyrobutyricum* ATCC 25755 genome. CTK_C05160 is the most likely *hydA* coding gene, and CTK_C029580 (*hyd*1) and CTK_C026290 (*hyd*2) belong to the group B family of monomeric hydrogenases. In previous studies, it has been reported that the *hydA* gene was impossible hardly to be knockout in the *C. acetobutylicum* [31, 32]. Therefore, this study firstly tried an inducible expression method to control *hydA* expression using a theophylline-dependent riboswitch (PfdxE) [33]. For evaluating the function of PfdxE in *C. tyrobutyricum*, a flavin mononucleotide-based fluorescent protein (BsFbFP) [34, 35] was used as a reporter fused with PfdxE. With increased theophylline concentrations to 8 mM, the fluorescence of the strain with PfdxE-BsFbFP showed a significant difference (p = 0.0011) compared with that without the inducer (Fig. 3A and Additional file 2: Fig. S3). Although the cultures were induced with 10 mM theophylline, the fluorescence intensity only showed a 0.33-fold increase compared with the non-induced culture. This result demonstrated that PfdxE could be used for inducible expression over a low dynamic range. In previous proteome analysis [25], hydA showed a low expression level in *C. tyrobutyricum*. Therefore, the characteristics of PfdxE perfectly fit the requirements for *hydA* expression regulation.

**Fig. 4:**
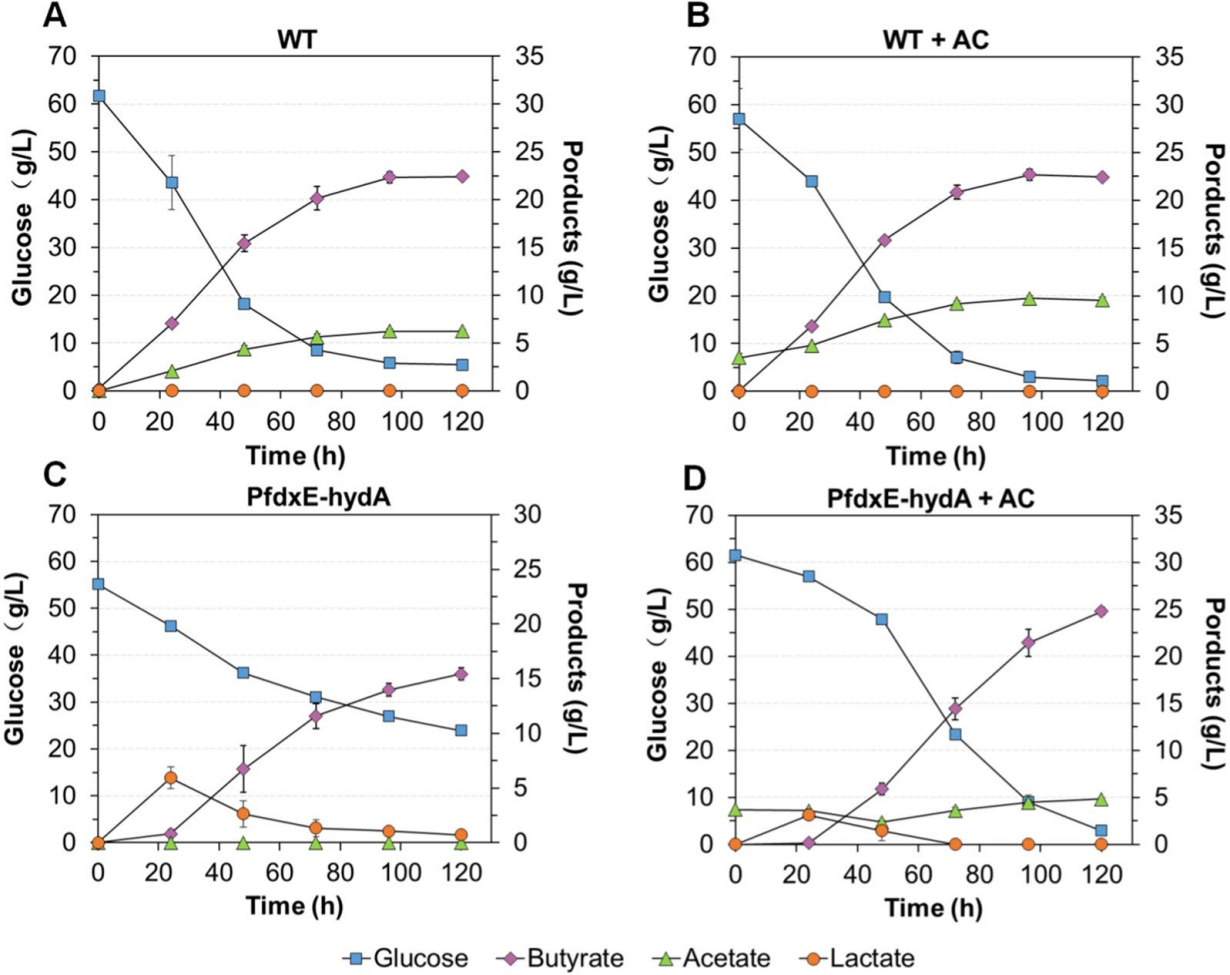
Fermentation performance of the wild-type and PfdxE-hydA strains in serum bottles. **A**, **B** show the fermentation kinetics of the wild-type and PfdxE-hydA strains, respectively. **C**, **D** show the fermentation kinetics under conditions of extra acetate supplementation of the wild-type and PfdxE-hydA strains, respectively. The values represent the data for the average of two replicates and the error bars represent the standard errors of these experiments

For regulating the HydA expression, the 691-bp upstream sequence of the *hydA* gene was replaced with PfdxE by using an endogenous CRISPR system [36]. Using PCR verification and PCR product sequencing (data not shown), the transformant was confirmed to be successfully modified and named as PfdxE-hydA (Fig. 3B and Additional file 2: Fig. S4). Then, the metabolic characteristics of PfdxE-hydA strain was evaluated in serum bottles. Without 10 mM theophylline culture, the growth of PfdxE-hydA showed a noticeable delay compared with the wild type (Fig. 3C). However, with 10 mM theophylline, the strain PfdxE-hydA reverted back to the original metabolic phenotype. Notably, over 0–48 h, acetate was undetectable in the culture of PfdxE-hydA without inducer, whereas lactate was detected under both the induced and non-induced conditions. To further compare the fermentation performance between PfdxE-hydA and the wild-type strain, a fermentation test was performed in serum bottles with 60 g/L glucose and 40 g/L CaCO_3_ as a pH buffer. During the fermentation (Fig. 4A and C), the PfdxE-hydA strain showed a distinctly slow metabolism, and lactate was also produced in the early stage, and then lactate was re-assimilated in the following fermentation. However, there was no acetate accumulation during the whole fermentation in PfdxE-hydA strain. Subsequently, an extra acetate supplementation fermentation (~ 5 g/L acetate) was carried out. There was a slight tendency to assimilate acetate at the early fermentation stage (Fig. 4B and D). However, acetate accumulation of PfdxE-hydA strain was distinctly inhibited by 81.8% compared with the wild type (Table 3). These results indicated that the HydA expression regulation was successful and inhibition of *hydA* expression could reduce acetate production. However, inhibiting HydA expression also weakened the ferredoxin redox loop (between the Hyds and Pfor), further restricted energy conversion. It could be the reason for the slow metabolism feature and activation of lactate synthesis. The activation of lactate synthesis could establish a new balance for cofactor turnover and energy conversion. The unsustained acetate assimilation could attribute to a compensation mechanism within Hyds expression, which can upregulate other Hyds (Hyd1 and Hyd2) for compensation. Thus, the PfdxE-hydA strain still produced 1.34 mM H_2_ under the non-induced conditions (Fig. 3C).

**Table 3:** Comparison of butyrate production using different strains and fermentation modes

Strain	Fermentation mode and pH strategy	Glucose consumed (g/L)	Acetate (g/L)	Lactate (g/L)	Butyrate		
					Titer (g/L)	Yield (g/g)^b^	BA/TA purity^a^ (%)
WT	Serum bottle with CaCO_3_ as a pH buffer	58.86 ± 0.77	6.75 ± 0.38	0	22.12 ± 0.00	0.38 ± 0.00	76.82 ± 0.76
WT + AC		54.79 ± 6.81	6.03 ± 0.16	0	22.33 + 0.54	0.41 ± 0.04	70.14 ± 0.24
PfdxE-hydA		31.18 ± 0.19	0.00 ± 0.00	0.72 ± 0.42	15.43 ± 0.55	0.49 ± 0.02	95.63 ± 2.34
PfdxE-hydA + AC		58.60 ± 0.61	1.10 ± 0.13	0	24.78 ± 0.39	0.42 ± 0.01	83.72 ± 0.22
ΔhydA		41.18 ± 4.80	2.46 ± 0.25	0	19.53 ± 1.41	0.48 ± 0.02	88.91 ± 1.48
Δhyd1		48.02 ± 3.45	5.00 ± 0.01	0	21.17 ± 0.26	0.44 ± 0.03	81.24 ± 0.15
Δhyd2		52.56 ± 2.33	5.74 ± 0.01	0	18.81 ± 0.14	0.36 ± 0.02	76.81 ± 0.19
WT	Bioreactor pH 5.5	52.43 ± 3.40	6.44 ± 1.01	0	17.62 ± 1.68	0.34 ± 0.01	73.30 ± 1.22
WT	Bioreactor pH .6.0	63.30 ± 3.16	6.89 ± 0.53	0	17.54 ± 1.65	0.29 ± 0.01	71.80 ± 0.33
WT	Bioreactor pH 6.5	50.19 ± 0.38	0.50 ± 0.11	6.53 ± 0.63	16.97 ± 0.52	0.34 ± 0.01	70.70 ± 2.16
ΔhydA	Bioreactor pH 5.5	44.68 ± 9.46	2.72 ± 2.24	0	18.25 ± 1.433	0.41 ± 0.05	87.75 ± 8.53
ΔhydA	Bioreactor pH 6.0	50.02 ± 0.86	2.89 ± 0.98	0	19.29 ± 1.50	0.39 ± 0.02	86.90 ± 4.71
ΔhydA ^c^	Bioreactor pH 6.5	45.60 ± 3.16	2.08 ± 0.02	12.32 ± 0.19	14.09 ± 1.76	0.31 ± 0.06	44.05 ± 2.72^c^

^a^BA/TA purity was calculated by the butyrate concentration divided by the total acid concentration, multiplied by 100%

^b^Yield based on glucose

^c^For the fermentation of the ΔhydA mutant at pH 6.5, there was 3.43 ± 0.05 g/L formate accumulation at the end of the fermentation. Therefore, the total acid concentration of the fermentation of the ΔhydA mutant at pH 6.5 included this concentration of formate

### Knockout of *hydA* resulted in upregulation of the gene expression of other Hyds

**Fig. 5:**
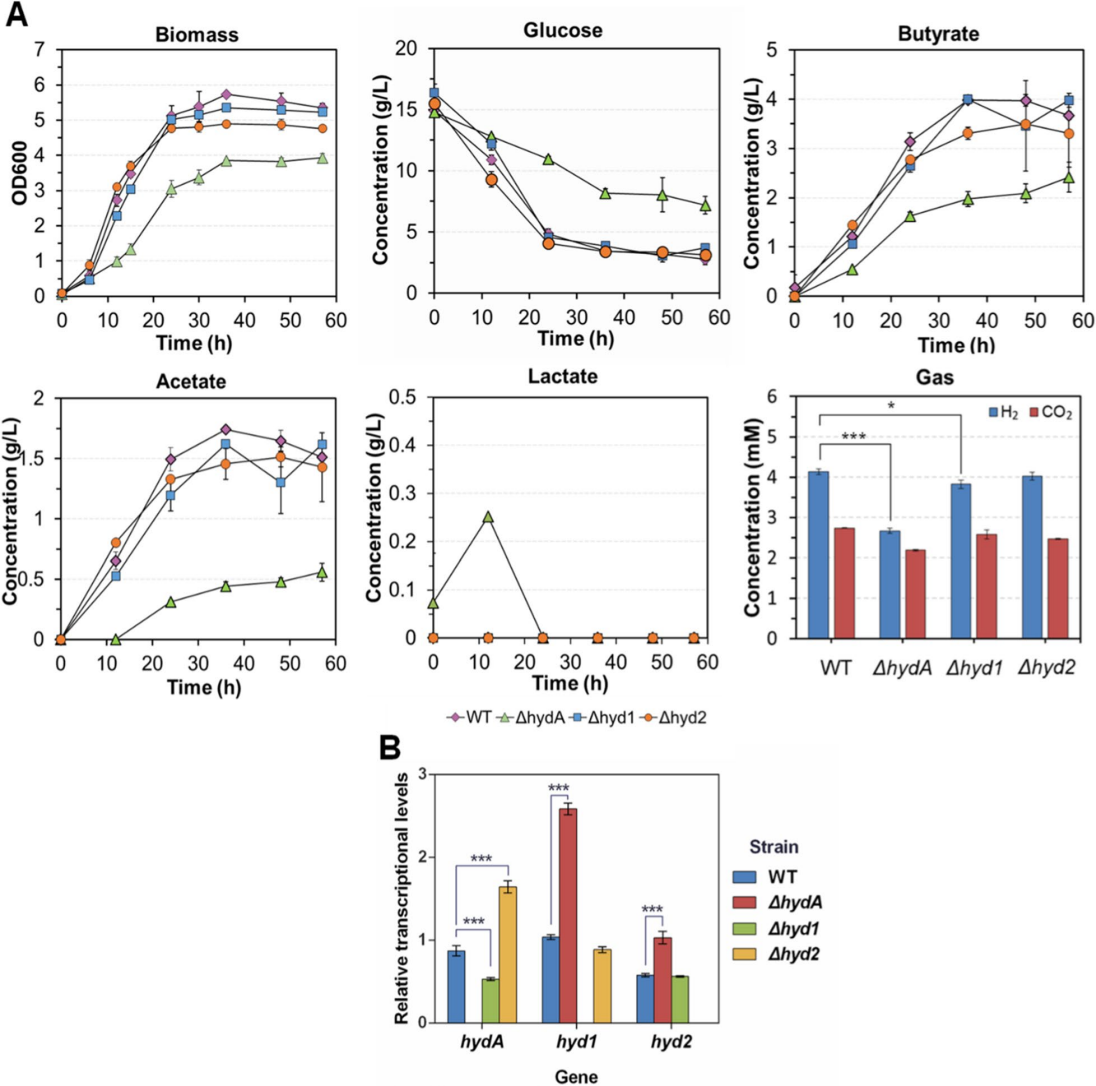
Effect of different Hyds knockout on the metabolic characteristics in *C. tyrobutyricum*. **A** Metabolic characteristics of different Hyd deficient strains under CGM culture conditions without CaCO_3_ as a pH buffer. **B** The relative transcriptional levels of *hydA*, *hyd*1 and *hyd*2 on the different strains. The values represent the averages of three replicates and the error bars represent the standard errors of these experiments (*p ≤ 0.05; **p ≤ 0.01; ***p ≤ 0.001, *t* test)

To further investigate the transcriptional compensation within Hyds and the effects of different Hyds knockout on the metabolic pattern, this study attempted to individually knockout *hydA*, *hyd*1, and *hyd*2. Almost all the coding sequence regions for these Hyds were deleted by the endogenous CRISPR system. PCR verification (Additional file 2: Fig. S5–7) and PCR product sequencing (data not shown) indicated that *hydA*, *hyd*1 and *hyd*2 were successfully deleted from the genome. These mutants indicated that individual Hyd gene deficiency did not lead to lethal effects in *C. tyrobutyricum* ATCC 25755. Then, in the metabolic characteristics evaluation for these mutants (Fig. 5A), the ΔhydA mutant (light green triangle) showed a distinctly slower metabolism and decreased H_2_ emission, which was similar to the results observed for the PfdxE-hydA strain without theophylline induction. However, the *Δhyd*1 and *Δhyd*2 mutants did not show any obvious change in metabolic characteristics, although the H_2_ emission of the *Δhyd*1 mutant showed a statistically significant decrease (*p* = 0.051) (Fig. 5A, Gas).

After analyzing the Hyds transcriptional changes in *ΔhydA*, *Δhyd*1 and *Δhyd*2 mutants, it was observed that *hyd*1 and *hyd*2 had a significant upregulation in* ΔhydA *mutant (Fig. 5B). In *Δhyd*2 mutant, the *hydA* gene was significantly upregulated. However, in *Δhyd*1 mutant, transcription of *hydA* and *hyd*2 genes showed no upregulation. These results demonstrated that HydA deficiency could induce a compensatory regulation by upregulation of both *hyd*1 and *hyd*2 in the transcription level. However, the *hyd*1 deficiency did not trigger compensatory regulation in the other *hyd* genes.

Fermentation performance of these mutants was also evaluated in serum bottles with 60 g/L glucose and 40 g/L CaCO_3_ as a pH buffer (Additional file 2: Fig. S8). The acetate accumulation of the *ΔhydA* mutant was reduced by 63.6% compared with the wild type (*ΔhydA*: 2.46 ± 0.25 g/L vs. WT: 6.75 ± 0.38 g/L). The butyrate yield and butyrate titer/acetate titer ratio (BA/AA) were increased by 26.3% (*ΔhydA*: 0.48 ± 0.02 g/g vs WT: 0.38 ± 0.00 g/g) and 15.7% (*ΔhydA*: 88.91 ± 1.48%vs. WT: 76.82 ± 0.76%), respectively. The *Δhyd*1 and *Δhyd*2 mutants did not show a distinct improvement in the butyrate yield or the BA/AA ratio (Table 3). However, the* ΔhydA* mutant did not appreciably produce lactate in the early stage of fermentation, which was different from the PfdxE-hydA strain (Fig. 4C). It could be due to Hyd compensation mechanism when HydA was completely deficient. Overall, inhibiting HydA can decrease acetate production and improve butyrate yield and BA/AA ratio in *C. tyrobutyricum*. It indicated that HydA played a critical role than other Hyds in *C. tyrobutyricum*. This phenomenon was also found in *C. acetobutylicum* ATCC 824 [32], but it was only observed in low pH (pH 5.0). The low pH will be beneficial to establish PMF because neutralophilic bacteria generally maintain their cytoplasmic pH in a neutral range (~7.5–7.7) [37]. Maintaining high PMF needs to improve proton efflux by the H_2_ emission (HydA), proton pump (RnfA-E), or the transportation of undissociated acid products. Therefore, with low pH, proton efflux by RnfA-E can be significantly enhanced by Hyd knockout, and then it led to the change in the distribution of products. Furthermore, in methanogenic archaeon, the energy conservation via hydrogen cycling, which generates proton motive force by coupling intracellular H_2_ emission and extracellular consumption, was also proposed to illustrate the energy generation mechanism [38]. In Methanosarcina barkeri, there are three types of energy-converting hydrogenases (the cytoplasmic F420-dependent, Frh; the energy-converting ferredoxin-dependent, Ech; the methanophenazine-dependent hydrogenase, Vht) [39]. These hydrogenases belong to the [NiFe] group of hydrogenases. It is quite different from *C. tyrobutyricum* (all are [Fe]-hydrogenase). The coupling mode should also have a big difference between methanogenic archaeon and *C. tyrobutyricum*. Therefore, in future studies, the specific functions and the contributions of different Hyds on PMF need to be further evaluated at the qualitative and quantitative levels.

### The effects of pH on the metabolic pattern

**Fig. 6:**
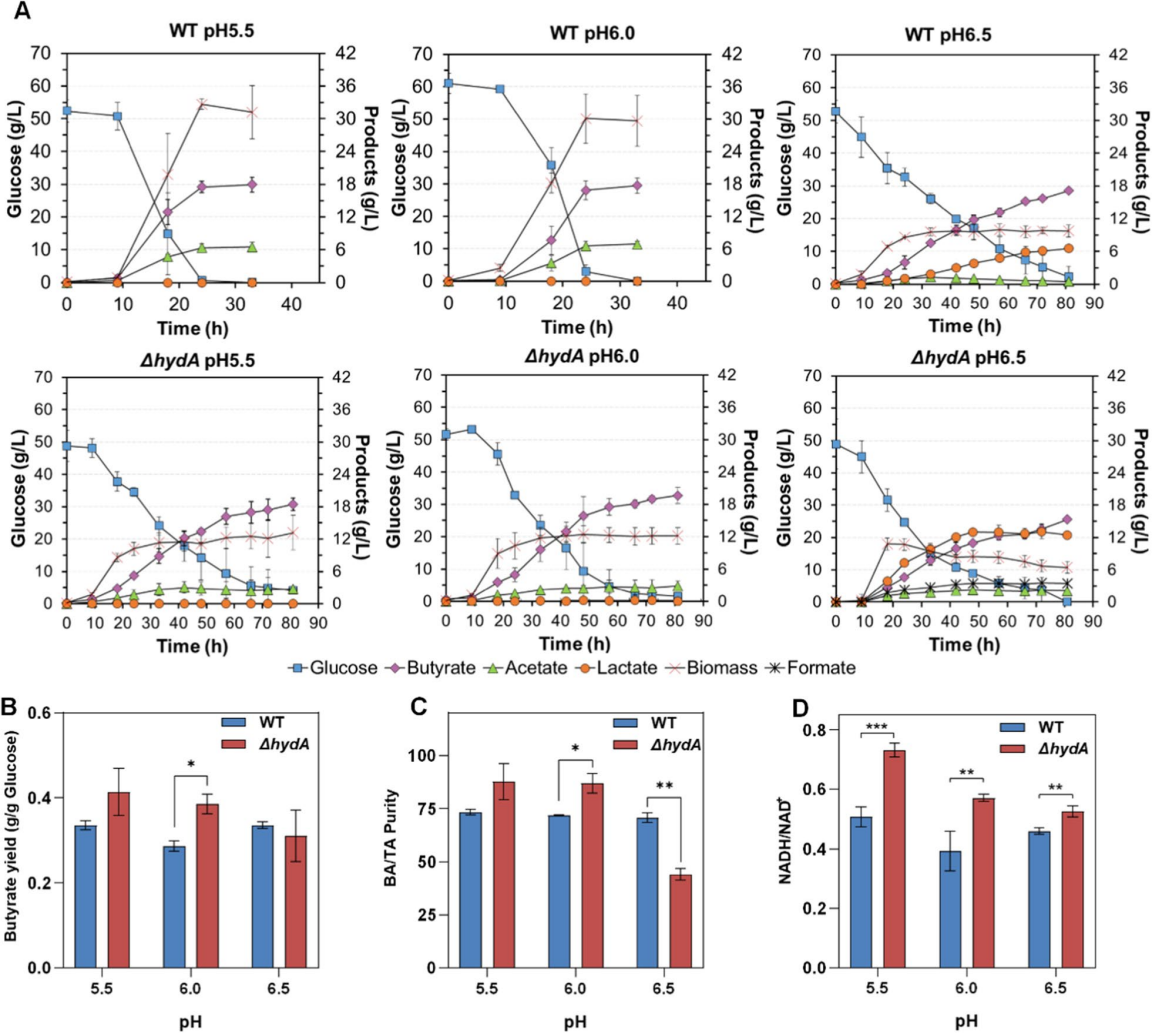
Comparison of the fermentation characteristics between the wild-type and *ΔhydA* strains under different pH in a stirred-tank bioreactor. **A** Fermentation kinetics of the wild-type and *ΔhydA* strains in CGM with glucose as a substrate. **B** Butyrate yields. **C** Butyrate purity (butyric acid titer/total acid titer, BA/TA). **D** NADH/NAD^+^ ratio in the wild-type and *ΔhydA* strains. The values represent the averages of three replicates and the error bars represent the standard errors of these experiments (*p ≤ 0.05; **p ≤ 0.01; ***p ≤ 0.001, *t* test)

For determining the effects of pH on the metabolic characteristic in* ΔhydA* mutant, this study also investigated the fermentation performance under three different pHs in a bioreactor. At pH 5.5 and 6.0 (Fig. 6A), the *ΔhydA* mutant showed a slow metabolic characteristic, and acetate accumulation was reduced by 57.8% and 58.1%, respectively, compared with the wild type (Table. 3). Furthermore, at pH 6.0, the butyrate yield and BA/TA rate for *ΔhydA* mutant were also increased by 34.5% and 21.0% (Fig. 6B, C) However, at pH 6.5, both the wild-type strain and the *ΔhydA* mutant showed an increased lactate production. In the *ΔhydA* mutant, lactate became a primary metabolic product (12.32 ± 0.19 g/L), and there was a distinct formate accumulation (3.43 ± 0.05 g/L). By evaluating the intracellular NADH/NAD^+^ ratio at the logarithmic stage of fermentation (Fig. 6D), the NADH/NAD^+^ ratios of the *ΔhydA* mutant showed a significant increase compared with those of the wild type (p_5.5_ = 0.00063, p_6.0_ = 0.0099, p_6.5_ = 0.0051). Notably, the gap in the NADH/NAD^+^ ratios between the *ΔhydA* mutant and the wild type became fewer as the pH increased. In general, intracellular pH was slightly higher than the extracellular pH in the neutralophilic bacterium [37]. For maintaining the PMF homeostasis, extracellular pH increase will trigger a higher intracellular pH. However, in our previous studies, we found that *C. tyrobutyricum* could not efficiently grow under an alkaline environment (pH > 7.0) (data not shown). It indicated that the higher intracellular pH could break through the tolerable pH range of *C. tyrobutyricum*. Therefore, once extracellular pH increases, a series of regulation for intracellular metabolism and physiological states change will form a new homeostasis PMF which was lower than that before pH increase. It meant that electrochemical potential from PMF could be not efficiently converted to redox potential. It is a kind of PMF dissipation in nature. The dissipation of PMF could further impair energy conversion and weaken the metabolic driving force for butyrate production. Essentially, activation of lactate production at higher pH was a metabolic characteristic under a status with low-energy conversion. When the *ΔhydA* mutant with pH 6.5, the formate accumulation could be attributed to the synergistic weakening both in a redox loop of ferredoxin (between Hyd and Pfor) and the PMF, which caused activation of pyruvate formate-lyase to compensate acetyl-CoA synthesis for cell growth. Therefore, the high-level intracellular NADH/NAD^+^ ratio in the *ΔhydA* mutant is certainly consistent with the status of in silico analysis, and the gap of NADH/NAD^+^ ratios between the *ΔhydA* mutant and the wild type becoming smaller with the pH also provide evidence for the coupling relationship between PMF and NADH turnover rate. Also, these results demonstrated that the energy conversion coupling driving mechanism is reasonable.

## Conclusion

Overall, this work provided the first GEM for *C. tyrobutyricum* ATCC 25755 (*i*CT583) and this model was capable of matching experimental growth and modeling the coupling metabolic behavior. Through this model, two redox loops of ferredoxin, which coupled the proton efflux with the processes of crotonyl-CoA reduction and pyruvate oxidation, were found to be the essential mechanisms of energy conversion coupling metabolic process. In this process, the electron transfer triggered by the redox reaction is converted into PFM, which supports energy supply for the basic process of cell activities. Then, the reaction products of the redox reaction as intermediate metabolites are further converted into end-products to maintain cellular homeostasis. Actually, for the *C. tyrobutyricum*, the products that human beings need were never the aim of cell lifecycle, but energy conversion for the reproduction. However, for biotransformation, efficient accumulation of desired products is the goal pursued by metabolic optimization. It will be an inevitable contradiction, unless the biotransformation is in the cell-free system. To address this challenge, deep understanding of metabolic driving mechanisms will be meaningful and useful for redesigning metabolism. This work revealed the energy conversion coupling driving mechanism for butyrate synthesis in *C. tyrobutyricum*, which can reasonably explain metabolic characteristics and phenomena in butyrate fermentation. Tuning this energy conversion system by regulating Hyd expression can significantly affect the distribution of fermentation products and metabolic characteristics. It demonstrated that the metabolic driving mechanism is an important factor for regulating metabolic flux. This insight can be further applied to metabolic optimization and develop *C. tyrobutyricum* as chassis cells for other chemicals and biofuel. In addition, this coupling driving mechanism also could be referenced for analyzing other Clostridium species, in which products synthesis was coupled with cellular growth.

## Materials and methods

### Construction of the GEM and metabolism network analysis

The genome sequence of *C. tyrobutyricum* ATCC 25755 was obtained from the work of Joungmin et al. [25]. Merlin software was used for re-annotating the genome sequence [40]. The COBRAToolbox 2.0 was used to read and convert the file into a stoichiometric matrix S in the MATLAB environment [41]. Gurobi, a linear programming solver, was used for performing flux balance analysis (FBA) [42], and a specific growth rate was set as the default objective function. The detailed processes of construction and refinement of the GEM are described in Additional file 2.

### Strains, media, and cultivation

Reinforced Clostridial Medium (RCM) (Hopebio, Tianjin, China) was used to activate *C. tyrobutyricum* ATCC 25755 at 37 °C under anaerobic conditions. The seed culture was cultured in a CGM medium for fermentation, and the composition is described in a previous study [16]. The seed culture was grown to OD_600_ = ~2.0 in 100-mL serum bottles containing 50 mL of liquid CGM, at 37 °C. For batch fermentation in serum bottles, the fermentation medium was based on the CGM medium supplemented with 60 g/L glucose and 40 g/L CaCO_3_ (for buffering pH changes). *Escherichia coli* Top10 was used as a cloning host for general plasmid construction. *E. coli* CA434 was used as the donor strain for conjugation [43]. *E. coli* were cultivated in Luria Broth (LB) medium at 37 °C (30 μg/mL chloramphenicol was supplied when necessary). Colonies of recombinant *C. tyrobutyricum* strains were screened on RCM plates (RCM medium with 20 g/L agar) supplemented with 250 μg/mL D-cycloserine and 30 μg/mL thiamphenicol.

### Plasmid construction and transformation

All plasmids and primers used in this study are shown in Additional file 1: Table. S2, S3. The CRISPR–Cas knockout plasmids were designed according to the method of Zhang et al. [36]. The detailed process of the plasmid construction is described in Additional file 2. The transfection of all knockout plasmids was performed by conjugation in an anaerobic environment. The protocol for the conjugation was as described in our previous study [18].

### Fermentation kinetics

The batch fermentation kinetics were studied in a 5-L bioreactor (Sartorius, Germany) with 1 L of CGM medium. Before inoculation, nitrogen gas was sparged in the bioreactor for ~ 30 min to reach anaerobic conditions. A 50 mL of the seed culture (5%) was used to inoculate the fermentation medium. After inoculation, the fermentation medium was agitated at 150 rpm and 37 °C, and the pH value was maintained using a 30% (v/v) ammonia solution. Samples were taken regularly to analyze the butyrate, acetate, residual sugar concentrations and the OD_600_ value.

### Evaluating expression level by fluorescence intensity

For detecting fluorescence intensity, the cells at the late logarithmic stage were collected by centrifuging at 5000 × g for 3 min, washed twice with PBS, and resuspended in the same-volume PBS buffer. Fluorescence intensity measurements were performed by a plate reader (SynergyMX, BioTek, Germany), with 150 μL volume for each sample in a 96-well plate. The wavelength excited was at 488 nm, and the wavelength captured was at 535 nm.

### Analytical methods

The glucose, butyrate and acetate concentrations were measured by HPLC (Waters, Milford, USA) using chromatographic conditions based on a previous protocol [6]. The cell density was measured using a spectrophotometer (PERSEE T6, Beijing, China). The biomass was determined by converting the OD_600_ value to a dry cell weight (DCW), which was converted as 1 OD_600_ = 0.30 ± 0.04 g DCW/L. The H_2_ and CO_2_ concentrations were measured by gas chromatography (Fuli 9790, Zhejiang, China) and the chromatographic conditions based on a previous protocol [44].

For intracellular NADH and NAD^+^ analysis, the wild type and ΔhydA mutant cells were collected after fermentation for 18 h in a bioreactor. The concentrations of NADH and NAD^+^ were measured using a NAD(H) content assay kit (Sangon Biotech, Shanghai, China). The NADH/NAD^+^ ratios were calculated from the concentration of NADH divided by the concentration of NAD^+^.

### Transcription level analysis

Total RNA was extracted using a bacteria RNA kit (TIANGEN, Beijing, China) and was reverse transcribed into cDNA using HiScript III RT SuperMix (Vazyme #R323-01, Nanjing, China). A relative quantitative PCR (qPCR) assay was performed using qPCR Master Mix (Vazyme #Q711, Nanjing, China). The specific primers used for qPCR are listed in Additional file 1: Table S2, and the translational GTPase coding gene (typA) was used as the reference gene.

## Supplementary Information

**Additional file 1: Table S1** Comparison of cell growth of *C. tyrobutyricum *in silico and in vivo^a^. **Table S2** Bacterial strains and plasmids used in this study. **Table S3** Primers used in this study.

**Additional file 2: Figure S1** Compared the arrangement of Bcd-EtfAB complex in the chromosome. *bcd*, butyryl-CoA dehydrogenase; *etfA*, electron transfer flavoprotein, alpha subunit; *etfB*, electron transfer flavoprotein, beta subunit; *hbd*, 3-hydroxybutyryl-CoA dehydrogenase; *crt*, 3-hydroxybutyryl-CoA dehydratase; *rex*, redox-sensitive transcriptional regulator. **Figure S2** CTR0446 (Hyd) and CTR0447 (RnfA-E) constraining simulation. In this process, FBA was used as the analysis method, and biomass synthesis reaction was set as the objective function. The reaction flux of CTR0446 and CTR0447 were constrained to increase gradually from 0 mmol/g DCW/h to 10 mmol/g DCW/h, respectively, then, the specific product rates of main productions were collected from every simulation result. **Figure S3** Characterization of theophylline riboswitches in *C. tyrobutyricum*. The values represent averages for three replicates and error bars represent standard errors of these experiments. (*p≤ 0.05; **p ≤ 0.01; ***p ≤0.001, *t *test). **Figure S4** Confirmation of replacement of the *hydA* promotor to theophylline-dependent inducible expression part. (A) Schematic of PCR verified method for PfdxE-hydA strain. Primers were marked as the red and blue arrows in (A), respectively, and the primer annealed site of hydA-test-F and hp-test-R were located at the flanks of the upper and lower homologous arms. (B)The agarose gel electrophoresis results for validating the PfdxE-hydA mutant. The 1 lane represented the PCR product of amplified using primers hydA-test-F and PfdxE-R; the 2 lane represented the PCR product amplified using primers PfdxE-F and hp-test-R. **Figure S5** Confirmation of knockout *hydA* gene. (A) Schematic of PCR verified method for* ΔhydA* strain. Primers were marked as the red arrow in (A), and the primers annealed site of T3-test-F and T3-test-R were located at the flanks of the upper and lower homologous arms. (B) The agarose gel electrophoresis results for PCR products. **Figure S6** Confirmation of knockout *hyd*1 gene. (A) Schematic of PCR verified method for *Δhyd*1 strain. Primers were marked as the red arrow in (A), and the primers annealed site of T5-test-F and T5-test-R were located at the flanks of the upper and lower homologous arms. (B) The agarose gel electrophoresis results of PCR products. **Figure S7** Confirmation of knockout *hyd*2 gene. (A) Schematic of PCR verified method for *Δhyd*2 strain. Primers were marked as the red arrow in (A), and the primers annealed site of T6-test-F and T6-test-R were located at the flanks of the upper and lower homologous arms. (B) The agarose gel electrophoresis results of PCR products. **Figure S8** Fermentation characteristic evaluation in serum bottles with CaCO_3_ as pH buffer. (A), (B), (C) and (D) represent wild-type strain, hydA deficient strain, *hyd*1 deficient strain, and *hyd*2 deficient strain, respectively. **Figure S9**. The phylogenetic tree of *C. tyrobutyricum* with relative organisms.

## Data Availability

All data generated or analyzed during this study are included in this published article and its Additional files.

## Abbreviations

GEM: Genome-scale model
HydA: Hydrogenase subunit A
RnfA-E: Rnf complex
PMF: Proton motive force
ORFs: Open reading frames
GAM: Growth-associated maintenance
NGAM: Non-GAM
PfdxE: Theophylline-dependent riboswitch
BsFbFP: Flavin mononucleotide-based fluorescent protein
BA/AA: Butyrate titer/acetate titer
FBA: Flux balance analysis
qPCR: Quantitative PCR
DCW: Dry cell weight
HPLC: High-performance liquid chromatography analysis
