# Supplementary material for: Model-based driving mechanism analysis for butyric acid production in Clostridium tyrobutyricum
Source: Biotechnol Biofuels Bioprod. 2022 Jun 25;15:71. doi: 10.1186/s13068-022-02169-z (PMC9233315; doi:10.1186/s13068-022-02169-z)
Supplement: Supplementary file 2 — Additional file 2: Figure S1 Compared the arrangement of Bcd-EtfAB complex in the chromosome. bcd, butyryl-CoA dehydrogenase; etfA, electron transfer flavoprotein, alpha subunit; etfB, electron transfer flavoprotein, beta subunit; hbd, 3-hydroxybutyryl-CoA dehydrogenase; crt, 3-hydroxybutyryl-CoA dehydratase; rex, redox-sensitive transcriptional regulator. Figure S2 CTR0446 (Hyd) and CTR0447 (RnfA-E) constraining simulation. In this process, FBA was used as the analysis method, and biomass synthesis reaction was set as the objective function. The reaction flux of CTR0446 and CTR0447 were constrained to increase gradually from 0 mmol/g DCW/h to 10 mmol/g DCW/h, respectively, then, the specific product rates of main productions were collected from every simulation result. Figure S3 Characterization of theophylline riboswitches in C. tyrobutyricum. The values represent averages for three replicates and error bars represent standard errors of these experiments. (*p≤ 0.05; **p ≤ 0.01; ***p ≤0.001, t test). Figure S4 Confirmation of replacement of the hydA promotor to theophylline-dependent inducible expression part. (A) Schematic of PCR verified method for PfdxE-hydA strain. Primers were marked as the red and blue arrows in (A), respectively, and the primer annealed site of hydA-test-F and hp-test-R were located at the flanks of the upper and lower homologous arms. (B)The agarose gel electrophoresis results for validating the PfdxE-hydA mutant. The 1 lane represented the PCR product of amplified using primers hydA-test-F and PfdxE-R; the 2 lane represented the PCR product amplified using primers PfdxE-F and hp-test-R. Figure S5 Confirmation of knockout hydA gene. (A) Schematic of PCR verified method for ΔhydA strain. Primers were marked as the red arrow in (A), and the primers annealed site of T3-test-F and T3-test-R were located at the flanks of the upper and lower homologous arms. (B) The agarose gel electrophoresis results for PCR products. Figure S6 Confirmation of k [file 13068_2022_2169_MOESM2_ESM.docx]

#### Additional file

#### Construction and Refinement of the GEM

In the preliminary the model construction stage, the *C. tyrobutyricum* ATCC25755 genome sequence was re-annotated using Merlin software to obtain the EC number and function annotation. After re-annotation, ~800 candidate genes were given an EC number. However, some candidate genes, such as those not involved in the biotransformation process or with nonholonomic EC numbers, and duplicate entries, needed to be manually verified. Finally, ~500 candidate genes were selected to assemble the draft model.

To maintain the accuracy of the annotation information, we manually revised the annotation entries by checking BRENDA (<https://www.brenda-enzymes.org/>), Uniprot (<http://www.uniprot.org/>), and KEGG (https://www.genome.jp/kegg/) databases. Some non-functional entries were eliminated because they could not be described in the metabolic network, such as reactions involving DNA or RNA duplication processes or signal transfer in transcription processes [[1](#_ENREF_1)]. For assembling the metabolic network, the EC numbers of genes were used as an associated label to collect corresponding reactions from a ModelSeed reaction database [[2](#_ENREF_2)]. The reaction direction, stoichiometry, transport, exchange, and demand (for filling gaps) reactions were verified and supplemented with information compared to with previously reported GEMs, in combination with literature data [[3-5](#_ENREF_3)]. The gapFind and checkBalance programs in the COBRA Toolbox were used to identify network gaps and unbalanced reactions [[6](#_ENREF_6)]. Thermodynamically infeasible loops were resolved by manually modifying the reaction directionality or removing unreasonable reactions caused by strained metabolic characteristics [[7](#_ENREF_7)].

We developed A phylogenetic tree to identify biomass composition and obtain appropriate information from closely related organisms. The 16S sequences of *C. tyrobutyricum* ATCC25755 and several closely related organisms were retrieved from the NCBI database (https://www.ncbi.nlm.nih.gov/) and analyzed using a neighbor-joining algorithm in MEGA X. As shown in Fig. S9, *C. tyrobutyricum* is closely related to *C. kluyveri* and *C. ljungdahlii*. The GEMs of *C. kluyveri* and *C. ljungdahlii* have been previously reported (Table.1). However, neither *i*CKL708 (*C. kluyveri*) nor *i*HN637 (*C. ljungdahlii*) have sufficient information regarding the biomass composition. For the model *i*CKL708, the biomass composition and coefficient were based on *Bacillus subtilis* [[8](#_ENREF_8)]. For *i*HN637, the biomass composition was derived from the model *i*CM925 (*C. beijerinckii*), which had a biomass composition similar to *C. acetobutylicum* [[3](#_ENREF_3)]. However, the major phospholipid content of *i*CM925 was determined from the Handbook on Clostridia [[5](#_ENREF_5)]. Therefore, in the present study, the biomass content of *C. tyrobutyricum* ATCC25755 is based on the model *i*CM925, consisting of DNA, RNA, lipids, proteins, peptidoglycans, teichoic acids, and trace metabolites [[3](#_ENREF_3), [5](#_ENREF_5)]. The DNA, RNA, and protein contents were identified from the genome sequence of *C. tyrobutyricum* ATCC25755 (Supplementary file1, Biomass) [[1](#_ENREF_1)], and the other contents were estimated similarly for the model *i*CM925 for *C. beijerincki* [[5](#_ENREF_5)]. The growth-associated maintenance (GAM) and non-GAM (NGAM) values were initially set as 40 and 5 mmol/g DCW/h, respectively, which were obtained from data for *C. acetobutlylicum* ATCC 824 [[3](#_ENREF_3)].

For qualitative evaluation, constrained FBA analysis was used to evaluate the integrity of the central metabolic pathway for metabolic carbon and nitrogen sources. The corresponding exchange reactions of the carbon or nitrogen sources were set as the sole carbon (with input flux at 10 mmol/g DCW/h) or nitrogen sources (with input flux at 1 mmol/g DCW/h) (Supplementary file1, Carbon and nitrogen), for filling the arabinose pathway, the reactions of arabinose isomerase (reaction ID: CTR0348), ribulokinase (CTR0789), and arabinose transporter (CTT0049), according to *i*Cac967 [[4](#_ENREF_4)]. The transport reactions (CTT0057 and CTT0058) as demand reactions were applied for filling glycerol and urea metabolic pathways.

In the quantitative evaluation, the batch fermentation of *C. tyrobutyricum* ATCC25755 with glucose in a 5-L bioreactor was performed, and the data from the exponential phase of the fermentation were used to refine the draft model. In the fitting simulation, the values for NGAM and GAM were set as 5 and 40 mmol ATP/g CDW/h, respectively, which referred to data of *C. acetobutylicum* [[3](#_ENREF_3)]. The concentrations of butyrate, acetate, and glucose were converted to the specific production and consumption rates as described by Thompson et al. [[9](#_ENREF_9)]. The specific production and consumption rates (Supplementary Table S1) were used as constraining conditions to simulate the specific growth rate by FBA analysis.

For other analyses, FBA was used to compute the maximum attainable growth rate with the glucose uptake set at 5 mmol/g DCW/h, GAM at 35 mmol/g DCW/h, and non-GAM at 5 mmol/g DCW/h (other constrained conditions are described in the following sections). The computational method for the flux-sum of the metabolites and the cofactor turnover rate is described in a previous study [[10](#_ENREF_10)].

#### Plasmid construction

For evaluating the characteristics of the theophylline-dependent expression part (PfdxE), the PfdxE fragment was synthetic by Genewiz Company (Suzhou, China). Then, the PfdxE fragment was fused with flavin mononucleotide–based fluorescent protein gene (*bsfbfp*) and cloned in pMTL82151 scaffold by Gibson Assembly (Seamless Assembly Cloning Kit, USA) to obtain plasmid of p82151-PfxdE-BsFbFP. For replaced the native promoter region of *hydA* with PfdxE, the p82151-gRNA-hydA-target1-PfdxE plasmid was constructed based on the pMTL82151 plasmid. Firstly, pMTL82151 scaffold was obtained by PCR amplified using the primer 82151-scaffold-F and 82151-scaffold-R. Then, the CRISPR transcriptional operon design referenced the study of J. Zhang et al [[11](#_ENREF_11)], in which spacer was replaced as spacer1 5’-AACAATTCAATATATGAATTTATATTATAACGTAGCTT-3’ targeting on the upstream of *hydA* gene coding sequence. For the construction of the CRISPR transcriptional operon, we firstly commissioned Genewiz company to synthetic the fragment of P*bgal*-repeat-spacer-repeat-terminator, which contain a complete CRISPR transcriptional operon. For the convenience of replacing spacer in following plasmid construction process, P*bgal*-repeat fragment and Repeat-terminator fragment was cloned in a pCloneEZ-Blunt-AMP plasmid (Clone Smarter, USA) to obtain pEZclone-P*bgal*-repeat and pEZclone-Repeat-terminator plasmid, respectively. The pEZclone-P*bgal*-repeat and pEZclone-Repeat-terminator plasmid were used as templates for amplifying Pbgal-repeat-spacer1 and Spacer1-repeat-terminator fragment, respectively. Then, the CRISPR transcriptional operon for spacer1 was obtained by fusing the Pbgal-repeat-spacer1 and the Spacer1-repeat-terminator fragments through overlap PCR. The CRISPR transcriptional operon for spacer1 was cloned in the pMTL82151 scaffold by gibson assembly to obtain the p82151-gRNA-hydA-target1 plasmid. Subsequently, theophylline-dependent expression parts (PfdxE) and two flanked homology arm sequences (~1 kb each) were cloned in linearization p82151-gRNA-hydA-target1 by *BssH* Ⅱ to obtain p82151-gRNA-hydA-target1-PfdxE. The PfdxE and homology arms were fused by overlapping PCR. Other knockout plasmids (p82151-gRNA-hydA-target3-HA, p82151-gRNA-hyd1-target6-HA, p82151-gRNA-hyd2-target5-HA) were constructed by a similar strategy of p82151-gRNA-hydA-target1-PfdxE, except that spacer and homology arms were replaced according to the need. Relevant characteristics of these plasmids were also described in Table S1. The primers for relative fragment amplifying were listed in Table S2.

### Additional file Figures

#### **Additional file Figure S1.** Compared the arrangement of Bcd-EtfAB complex in the chromosome. *bcd*, butyryl-CoA dehydrogenase; *etfA*, electron transfer flavoprotein, alpha subunit; *etfB*, electron transfer flavoprotein, beta subunit; *hbd*, 3-hydroxybutyryl-CoA dehydrogenase; *crt*, 3-hydroxybutyryl-CoA dehydratase; *rex*, redox-sensitive transcriptional regulator.


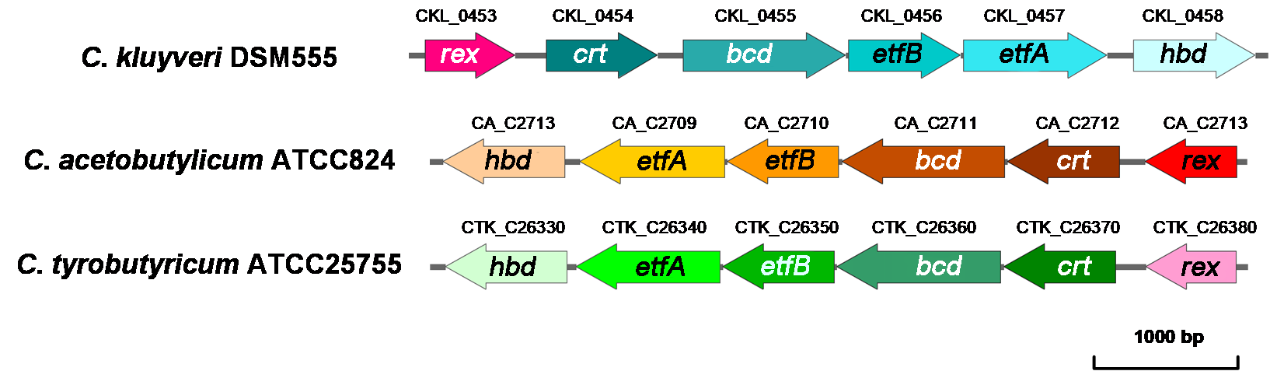


#### Additional file Figure S2. CTR0446 (Hyd) and CTR0447 (RnfA-E) constraining simulation. In this process, FBA was used as the analysis method, and biomass synthesis reaction was set as the objective function. The reaction flux of CTR0446 and CTR0447 were constrained to increase gradually from 0 mmol/g DCW/h to 10 mmol/g DCW/h, respectively, then, the specific product rates of main productions were collected from every simulation result.


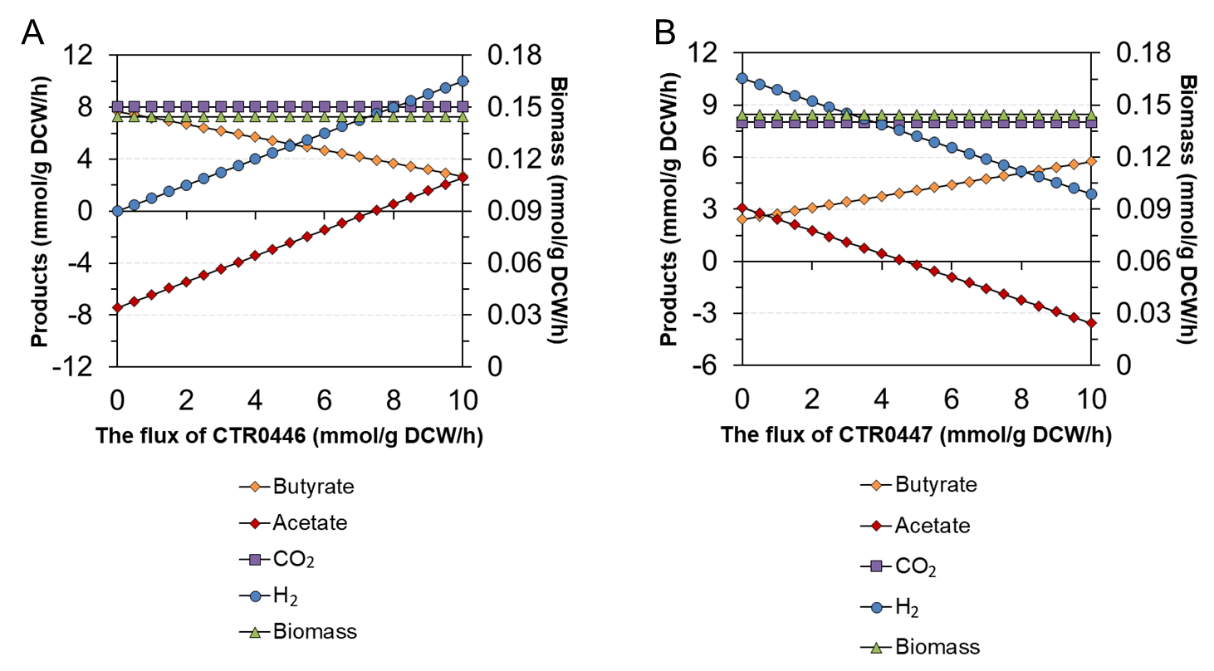


#### Additional file Figure S3. Characterization of theophylline riboswitches in *C. tyrobutyricum*. The values represent averages for three replicates and error bars represent standard errors of these experiments. (**p*≤ 0.05; ***p* ≤ 0.01; ****p* ≤0.001, *t* test)


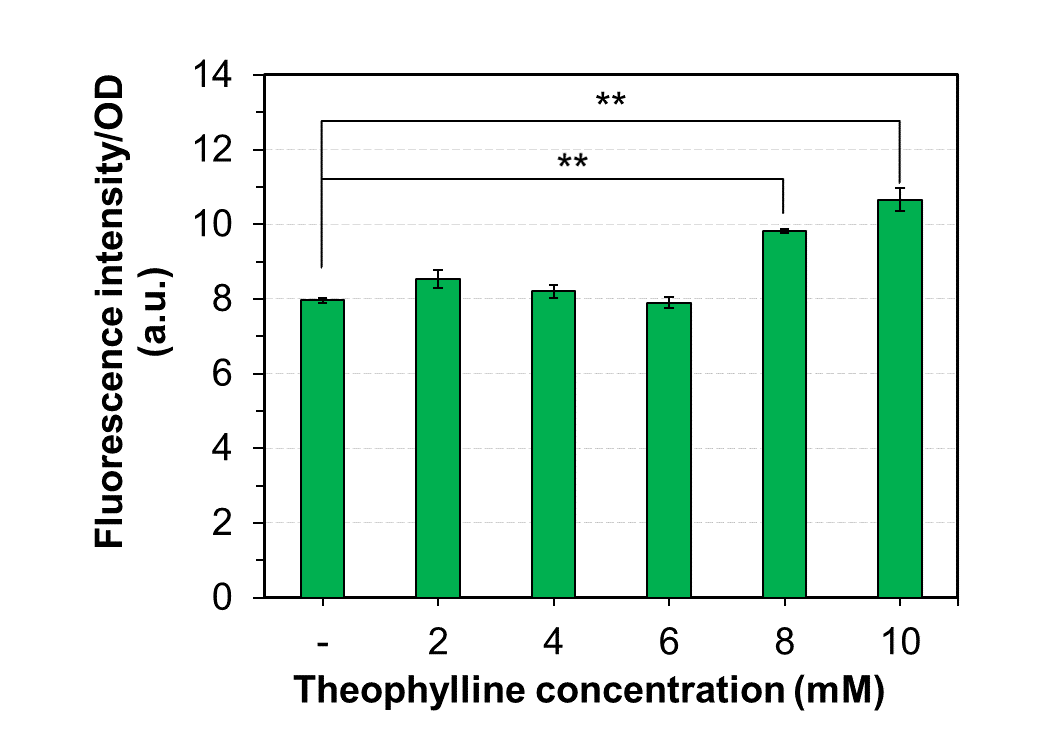


#### Additional file Figure S4. Confirmation of replacement of the *hydA* promotor to theophylline-dependent inducible expression part. (A) Schematic of PCR verified method for PfdxE-hydA strain. Primers were marked as the red and blue arrows in (A), respectively, and the primer annealed site of hydA-test-F and hp-test-R were located at the flanks of the upper and lower homologous arms. (B)The agarose gel electrophoresis results for validating the PfdxE-hydA mutant. The 1 lane represented the PCR product of amplified using primers hydA-test-F and PfdxE-R; the 2 lane represented the PCR product amplified using primers PfdxE-F and hp-test-R.

#### Additional file Figure S5. Confirmation of knockout *hydA* gene. (A) Schematic of PCR verified method for *ΔhydA* strain. Primers were marked as the red arrow in (A), and the primers annealed site of T3-test-F and T3-test-R were located at the flanks of the upper and lower homologous arms. (B) The agarose gel electrophoresis results for PCR products.


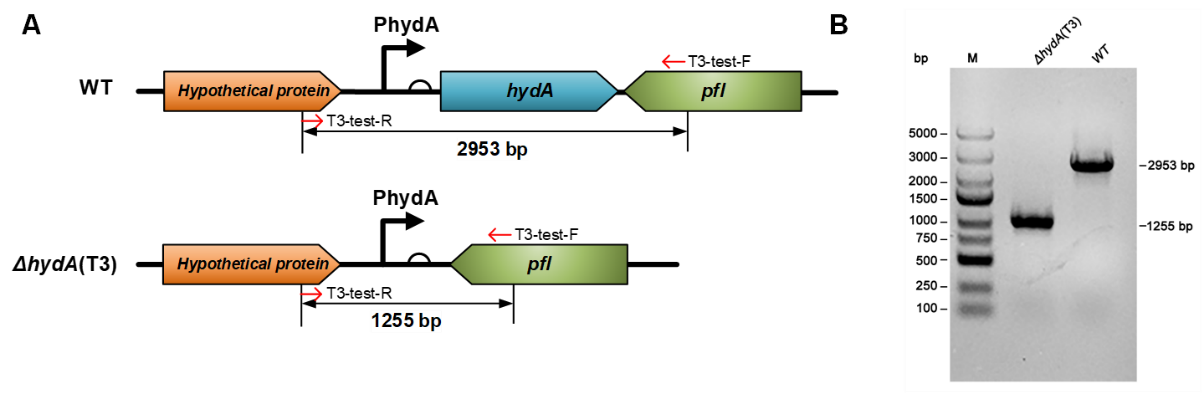


#### Additional file Figure S6. Confirmation of knockout *hyd1* gene. (A) Schematic of PCR verified method for *Δhyd1* strain. Primers were marked as the red arrow in (A), and the primers annealed site of T5-test-F and T5-test-R were located at the flanks of the upper and lower homologous arms. (B) The agarose gel electrophoresis results of PCR products.

#### Additional file Figure S7. Confirmation of knockout *hyd2* gene. (A) Schematic of PCR verified method for *Δhyd2* strain. Primers were marked as the red arrow in (A), and the primers annealed site of T6-test-F and T6-test-R were located at the flanks of the upper and lower homologous arms. (B) The agarose gel electrophoresis results of PCR products.


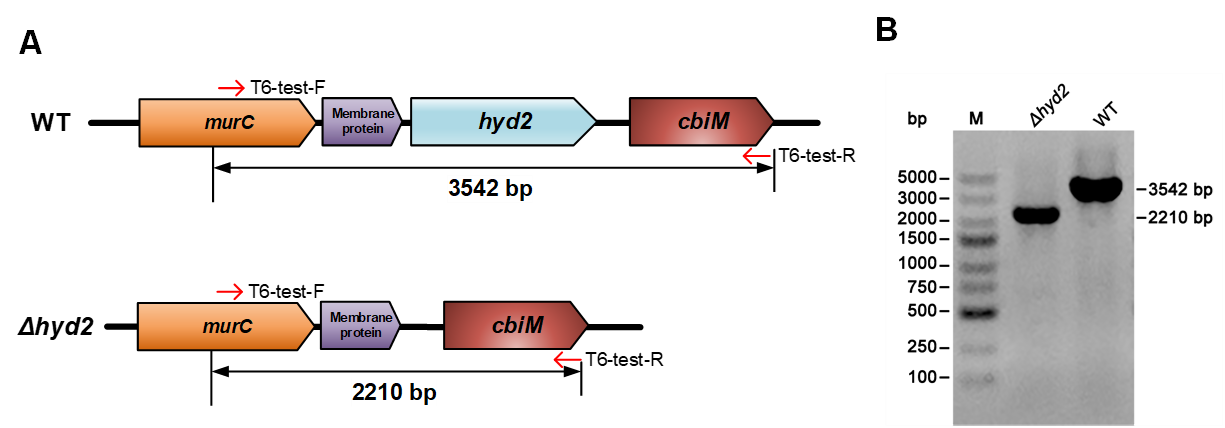


#### **Additional file Figure S8.** Fermentation characteristic evaluation in serum bottles with CaCO_3_ as pH buffer. (A), (B), (C) and (D) represent wild-type strain, *hydA* deficient strain, *hyd1* deficient strain, and *hyd2* deficient strain, respectively.

#### Additional file **Figure S9**. The phylogenetic tree of *C. tyrobutyricum* with relative organisms.

*
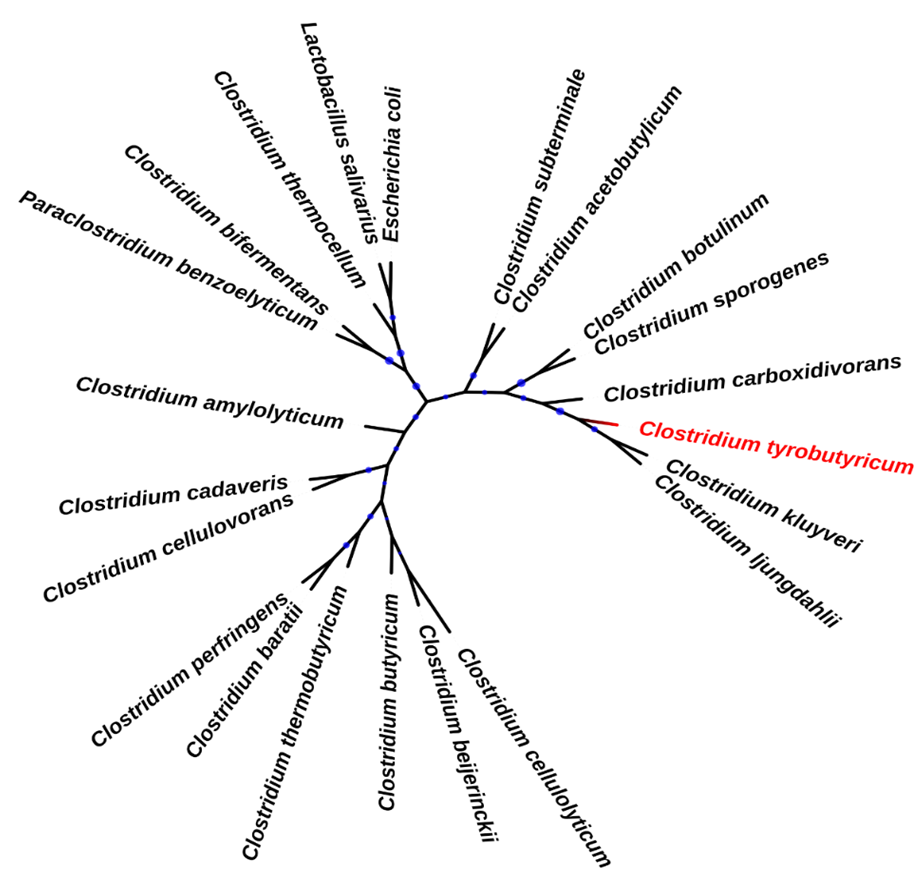
*

### Additional file Tables

#### Table S1. Comparison of cell growth of *C. tyrobutyricum in silico* and *in vivo*^a^

| Fermentation mode | GAM | NGAM | Specific growth  rate (g DCW·h^-1^) | Constraint condition  (mmol/g DCW/h) | | | Reference |
| --- | --- | --- | --- | --- | --- | --- | --- |
|  | mmol ATP/g DCW/h | |  | Glucose | Butyrate | Acetate |  |
| Simulation | 40 | 5 | 0.17 | 5.88 | 3.65 | 1.97 | This study |
| Simulation | 35 | 5 | 0.18 | 5.88 | 3.60 | 1.97 | This study |
| Batch | - | - | 0.20 ± 0.05 | 5.88 ± 0.81 | 3.80 ± 1.09 | 1.97 ± 0.44 | This study |
| Fed-batch | - | - | 0.063 ± 0.00 | - | - | - | [[12](#_ENREF_12)] |
| Fed-batch | - | - | 0.21 ± 0.04 | - | - | - | [[13](#_ENREF_13)] |
| Fed-batch | - | - | 0.28 ± 0.03 | - | - | - | [[14](#_ENREF_14)] |
| In serum tubes | - | - | 0.178 ± 0.01 | - | - | - | [[15](#_ENREF_15)] |
| Batch | - | - | 0.208 ± 0.00 | - | - | - | [[16](#_ENREF_16)] |
| Batch | - | - | 0.19 ± 0.01 | - | - | - | [[17](#_ENREF_17)] |
| Batch | - | - | 0.15 ± 0.02 | - | - | - | [[18](#_ENREF_18)] |
| Batch | - | - | 0.16 ± 0.01 | - | - | - | [[19](#_ENREF_19)] |

*^a^* The substrate uptake and product secretion rates were constrained by referring to the measured value of the exponential growth phase.

#### Table S2. Bacterial strains and plasmids used in this study

| **Strains** | **Relevant characteristics** | **Reference/Source** |
| --- | --- | --- |
| WT | *C. tyrobutyricum* ATCC 25755 | ATCC |
| PfdxE-BsFbFP | *C. tyrobutyricum* ATCC 25755 containing p82151-PfxdE-BsFbFP | This study |
| BsFbFP | *C. tyrobutyricum* ATCC 25755 containing p82151- BsFbFP | This study |
| PfdxE-hydA | Derived from ATCC 25755, with the native promoter region of *hydA* replaced with PfdxE | This study |
| *ΔhydA* | Derived from ATCC 25755, with *hydA* coding sequence deleted (from position -7 to +1691)(If not specified, the following defaults the +1 position is the first base of the initiation codon as +1 position) | This study |
| *Δhyd1* | Derived from ATCC 25755, with *hyd1* coding sequence deleted (from position +1 to +1332) | This study |
| *Δhyd2* | Derived from ATCC 25755, with *hyd2* coding sequence deleted (from position +1 to +1488) | This study |
| *E. coli* DH5α | Host cells for plasmid preparation | TIANJIN, China |
| *E. coli* CA434 | Donor cells for conjugation transformation | [[20](#_ENREF_20)] |
| **Plasmid** | **Relevant characteristics** | **Reference/Source** |
| pMTL82151 | ColE1 ori; CmR; pBP1 ori, TraJ | [[21](#_ENREF_21)] |
| p82151-PfxdE-BsFbFP | pMTL82151 derivative, a theophylline-dependent expression part (PfxdE) was used to drive the expression of flavin mononucleotide–based fluorescent proteins (BsFbFP) | This study |
| p82151-BsFbFP | pMTL82151 derivative, no promoter drive BsFbFP | This study |
| pCloneEZ-Blunt-AMP | ColE1 ori, AmpR | Clone Smarter, USA |
| pEZclone-Pbgal-repeat | pCloneEZ-Blunt-AMP derivative, Pbgal-repeat | This study |
| pEZclone-Repeat-terminator | pCloneEZ-Blunt-AMP derivative, Repeat-terminator | This study |
| p82151-gRNA-hydA-target1-PfdxE | pMTL82151 derivative, A lactose inducible promoter (Pbgal) was used to drive the transcription of spacer1 (5’- AACAATTCAATATATGAATTTATATTATAACGTAGCTT -3’) targeting on the upstream of *hydA* gene coding sequence, and two homology arms (~1 kb each) for the replaced native promoter region of *hydA* with PfdxE | This study |
| p82151-gRNA-hydA-target3-HA | Same as p82151-gRNA-hydA-target1-PfdxE, except that the spacer was replaced with spacer3 (5’- CCACAGCAGATATTCGGTACTGCCACTAAAACCTATTA-3’) and two homology arms (~1 kb each) for *hydA* gene coding sequence deletion. | This study |
| p82151-gRNA-hyd1-target6-HA | Same as p82151-gRNA-hydA-target3, except that the spacer3 was replaced with spacer6 (5’-TCTTCACTATTTACGTAATGATCGAATTCTATGGATTC-3’) and two homology arms (~1 kb each) for *hyd1* gene coding sequence deletion. | This study |
| p82151-gRNA-hyd2-target5-HA | Same as p82151-gRNA-hydA-target3, except that the spacer3 was replaced with spacer5 (5’- GAAGTTATGAGACCATGTAAGAAGTCATGTCCTACAGG-3’) and two homology arms (~0.5 kb each) for *hyd2* gene coding sequence deletion. | This study |

#### Table S3. Primers used in this study

| **Primers** | **Sequences (5′-3′)** | **Description** |
| --- | --- | --- |
| 82151-scaffold-F | ggcgcgccgttctgaatcct | Amplification of pMTL82151 scaffold |
| 82151-scaffold-R | tcgactctagaggatccccgg |  |
| BsFbFP-F | atggctagttttcaaagttttg | Amplification of the *Bsfbfp* gene |
| BsFbFP-R | ccggggatcctctagagtcgattacattataggtaatacttttacagc |  |
| PfxdE-BsFbFP-F | aaactttgaaaactagccatcttgttgttacctccttagcagggtgctgc | Amplification of PfdxE |
| PfxdE-p8-R | aggattcagaacggcgcgcctacagttatataaaaattac |  |
| Pbgal-F-2 | ccggggatcctctagagtcgagttatatacttggtttatttacttg | Amplification of Pbgal-repeat-spacer1 fragment |
| Pbgal-t1-R | aagctacgttataatataaattcatatattgaattgttatttaaatacatctcatgtt |  |
| Pbgal-t1-F | aacaattcaatatatgaatttatattataacgtagcttgttgaaccttaacatgagatg | Amplification of spacer1-repeat-terminator fragment |
| Repeat-terminator-p8-R | aggattcagaacggcgcgccaccatgattacgccaagc |  |
| Terminator-hydA-F | gcgtaatcatggtggcgcgcctttcgcttctatccccgat | Amplification of upstream homologous arm for the replaced native promoter region of *hydA* with PfdxE |
| HydA-PfdxE-R | gcagcaccctgctaaggaggtaacaacaagatgattacagttgttttaga |  |
| PfxdE-F | cctccttagcagggtgctgc | Amplification of the PfdxE for linking to homologous arm |
| PfdxE-R | cattttgtgcagtaattcattacagttatataaaaattac |  |
| PfxdE -hydA-F | atgaattactgcacaaaatg | Amplification of downstream homologous arm for the replaced native promoter region of *hydA* with PfdxE |
| hydA-p8-R | aggattcagaacggcgcgcatttattacctccagaataaaatttc |  |
| Pbgal-t3-R | ttagtggcagtaccgaatatctgctgtggatttaaatacatctcatgtt | With primer of Pbgal-F-2, amplification of Pbgal-repeat-spacer3 fragment |
| Pbgal-t3-F | atattcggtactgccactaaaacctattagttgaaccttaacatgagat | With primer of Repeat-terminator-p8-R, amplification of spacer3-repeat-terminator fragment |
| T3-arm-up-F | gcgtaatcatggtggcgcgctgaatcgtggatttgacagt | Amplification of upstream homologous arm for *hydA* gene coding sequence deletion |
| T3-arm-up-R2 | tatttaaaaggagtatttaagctgcttcatcataactatgc |  |
| T3-arm-down-F2 | ttaaatactccttttaaataatcaaattaag | Amplification of downstream homologous arm for *hydA* gene coding sequence deletion |
| T3-arm-down-R | aggattcagaacggcgcgcttagagtaaagtatcttgctcag |  |
| Pbgal-T6-R | gaatccatagaattcgatcattacgtaaatagtgaagaatttaaatacatctcatgtt | With primer of Pbgal-F-2, amplification of Pbgal-repeat-spacer6 fragment |
| Pbgal-T6-F | tcttcactatttacgtaatgatcgaattctatggattcgttgaaccttaacatgagat | With primer of Repeat-terminator-p8-R, amplification of spacer6-repeat-terminator fragment |
| T6-up-arm-F | gcgtaatcatggtggcgcgccattaatattaaatattcccgatac | Amplification of upstream homologous arm for *hyd1* gene coding sequence deletion |
| T6-up-arm-R | gtagtttaaaagttcctctctccaaacagctcctcgggaa |  |
| T6-down-arm-F | gagaggaacttttaaactac | Amplification of downstream homologous arm for *hyd1* gene coding sequence deletion |
| T6-down-arm-R | aggattcagaacggcgcgccaccttctgctatagctaga |  |
| Pbgal-T5-R | cctgtaggacatgacttcttacatggtctcataacttcatttaaatacatctcatgtt | With primer of Pbgal-F-2, amplification of Pbgal-repeat-spacer5 fragment |
| Pbgal-T5-F | gaagttatgagaccatgtaagaagtcatgtcctacagggttgaaccttaacatgagat | With primer of Repeat-terminator-p8-R, amplification of spacer5-repeat-terminator fragment |
| T5-down-arm3-F | gcgtaatcatggtggcgcgcggatactgtatatgcaattacac | Amplification of downstream homologous arm for *hyd2* gene coding sequence deletion |
| T5-down-arm3-R | tatattgtgagaacagcttaa |  |
| T5-up-arm3-F | taagctgttctcacaatatacaataatttaagtatgattcc | Amplification of upstream homologous arm for *hyd2* gene coding sequence deletion |
| T5-up-arm3-R | aggattcagaacggcgcgccgtatagtctatttaaaatggag |  |
| Test-ct-F | gcgttttcttcagcttcta | For validating the strain of native *hydA* promoter region replaced to PfdxE |
| Test-ct-R | ttatggcatgtggtttcac |  |
| hydA-test-F | gctcccgcaccactgtat |  |
| PfdxE-R | cattttgtgcagtaattcattacagttatataaaaattac |  |
| PfxdE-F | cctccttagcagggtgctgc |  |
| hp-test-R | tacagtaaacaccgtcaa |  |
| T3-up-test2-F | gtccattgatgacgccagag | For validating the strain of *hydA* gene coding sequence deletion |
| hydA-test2-R | gctctacatcactatctccc |  |
| T6-test-F | aacagcggagatgtaatagc | For validating the strain of *hyd1* gene coding sequence deletion |
| T6-test-R | ttaaatagcctccttgtctt |  |
| T5-test-F | ctgtttccaattaaatatcaa | For validating the strain of *hyd2* gene coding sequence deletion |
| T5-test-R | atatattccatgggcattc |  |
| *hydA*-qPCR-F1 | gaccttgacctgggtttcc | RT-qPCR amplification of the *hydA* gene |
| *hydA* -qPCR-R1 | agtcctgagggcttctgtt |  |
| *hyd1* -qPCR-F1 | agcaccagcaatagtaggg | RT-qPCR amplification of the *hyd1* gene |
| *hyd1* -qPCR-R1 | tgtgagcatatctgcgaaa |  |
| *hyd2* -qPCR-F1 | tagccgcagtaactccacc | RT-qPCR amplification of the *hyd2* gene |
| *hyd2* -qPCR-R1 | tgtatttgtagggccttgt |  |
| *typA*-F | caataattgcccatgtagatcacgg | RT-qPCR amplification of the *typA* gene |
| *typA*-R | gagtccataaccctctcttgaac |  |

#### SUPPLEMENTARY REFERENCES

1. Thiele I, Palsson BO: A protocol for generating a high-quality genome-scale metabolic reconstruction. *Nat Protoc* 2010, 5(1):93-121.

2. Devoid S, Overbeek R, DeJongh M, Vonstein V, Best AA, Henry C: Automated genome annotation and metabolic model reconstruction in the SEED and Model SEED. *Methods in molecular biology (Clifton, NJ)* 2013, 985:17-45.

3. Lee J, Yun H, Feist AM, Palsson BØ, Lee SY: Genome-scale reconstruction and in silico analysis of the *Clostridium acetobutylicum* ATCC 824 metabolic network. *Appl Microbiol Biotechnol* 2008, 80(5):849-862.

4. Yoo M, Bestel-Corre G, Croux C, Riviere A, Meynial-Salles I, Soucaille P: A Quantitative System-Scale Characterization of the Metabolism of *Clostridium acetobutylicum*. *Mbio* 2015, 6(6):12.

5. Milne CB, Eddy JA, Raju R, Ardekani S, Kim PJ, Senger RS, Jin YS, Blaschek HP, Price ND: Metabolic network reconstruction and genome-scale model of butanol-producing strain *Clostridium beijerinckii* NCIMB 8052. *BMC Syst Biol* 2011, 5:15.

6. Schellenberger J, Que R, Fleming RM, Thiele I, Orth JD, Feist AM, Zielinski DC, Bordbar A, Lewis NE, Rahmanian S *et al*: Quantitative prediction of cellular metabolism with constraint-based models: the COBRA Toolbox v2.0. *Nat Protoc* 2011, 6(9):1290-1307.

7. Schellenberger J, Lewis NE, Palsson BO: Elimination of Thermodynamically Infeasible Loops in Steady-State Metabolic Models. *Biophysical Journal* 2011, 100(3):544-553.

8. Zou W, Ye GB, Zhang J, Zhao CQ, Zhao XX, Zhang KZ: Genome-scale metabolic reconstruction and analysis for *Clostridium kluyveri*. *Genome* 2018, 61(8):605-613.

9. Thompson RA, Dahal S, Garcia S, Nookaew I, Trinh CT: Exploring complex cellular phenotypes and model-guided strain design with a novel genome-scale metabolic model of *Clostridium thermocellum* DSM 1313 implementing an adjustable cellulosome. *Biotechnol Biofuels* 2016, 9.

10. Feng J, Yang J, Li X, Guo M, Wang B, Yang ST, Zou X: Reconstruction of a genome-scale metabolic model and in silico analysis of the polymalic acid producer *Aureobasidium pullulans* CCTCC M2012223. *Gene* 2017, 607:1-8.

11. Zhang J, Zong W, Hong W, Zhang Z-T, Wang Y: Exploiting endogenous CRISPR-Cas system for multiplex genome editing in *Clostridium tyrobutyricum* and engineer the strain for high-level butanol production. *Metab Eng* 2018, 47:49-59.

12. Zhu Y, Yang ST: Adaptation of *Clostridium tyrobutyricum* for enhanced tolerance to butyric acid in a fibrous-bed bioreactor. *Biotechnol Prog* 2003, 19(2):365-372.

13. Liu XG, Yang ST: Kinetics of butyric acid fermentation of glucose and xylose by *Clostridium tyrobutyricum* wild type and mutant. *Process Biochem* 2006, 41(4):801-808.

14. Liu XG, Zhu Y, Yang ST: Butyric acid and hydrogen production by *Clostridium tyrobutyricum* ATCC 25755 and mutants. *Enzyme Microb Technol* 2006, 38(3-4):521-528.

15. Jiang L, Wang J, Liang S, Cai J, Xu Z, Cen P, Yang S, Li S: Enhanced Butyric Acid Tolerance and Bioproduction by *Clostridium tyrobutyricum* Immobilized in a Fibrous Bed Bioreactor. *Biotechnol Bioeng* 2011, 108(1):31-40.

16. Yu M, Zhang Y, Tang IC, Yang S-T: Metabolic engineering of *Clostridium tyrobutyricum* for n-butanol production. *Metab Eng* 2011, 13(4):373-382.

17. Yu M, Du Y, Jiang W, Chang W-L, Yang S-T, Tang IC: Effects of different replicons in conjugative plasmids on transformation efficiency, plasmid stability, gene expression and n-butanol biosynthesis in *Clostridium tyrobutyricum*. *Appl Microbiol Biotechnol* 2012, 93(2):881-889.

18. Zhang YL, Yu MR, Yang ST: Effects of ptb knockout on butyric acid fermentation by *Clostridium tyrobutyricum*. *Biotechnol Prog* 2012, 28(1):52-59.

19. Fu H, Yu L, Lin M, Wang J, Xiu Z, Yang S-T: Metabolic engineering of *Clostridium tyrobutyricum* for enhanced butyric acid production from glucose and xylose. *Metab Eng* 2017, 40:50-58.

20. Williams DR, Young DI, Young M: Conjugative plasmid transfer from Escherichia coli to *Clostridium acetobutylicum*. *J Gen Microbiol* 1990, **136**(5):819-826.

21. Heap JT, Pennington OJ, Cartman ST, Minton NP: **A modular system for Clostridium shuttle plasmids**. *J Microbiol Methods* 2009, **78**(1):79-85.
